# The atypical Rho GTPase Rnd2 is critical for dentate granule neuron development and anxiety-like behavior during adult but not neonatal neurogenesis

**DOI:** 10.1038/s41380-021-01301-z

**Published:** 2021-09-24

**Authors:** Thomas Kerloch, Fanny Farrugia, Lou Bouit, Marlène Maître, Geoffrey Terral, Muriel Koehl, Pierre Mortessagne, Julian Ik-Tsen Heng, Mylène Blanchard, Hélène Doat, Thierry Leste-Lasserre, Adeline Goron, Delphine Gonzales, David Perrais, François Guillemot, Djoher Nora Abrous, Emilie Pacary

**Affiliations:** 1 Univ. Bordeaux, INSERM, Neurocentre Magendie, U1215, F-3300 Bordeaux, France; 2 Univ. Bordeaux, CNRS, Interdisciplinary Institute for Neuroscience, IINS, UMR 5297, F-33000 Bordeaux, France; 3 Laser microdissection Facility, Univ. Bordeaux, INSERM, Neurocentre Magendie, U1215, F-3300 Bordeaux, France; 4 Curtin Health Innovation Research Institute, Curtin University, 6102 Bentley, WA Australia; 5 Transcriptome Facility, Univ. Bordeaux, INSERM, Neurocentre Magendie, U1215, F-3300 Bordeaux, France; 6 Genotyping Facility, Univ. Bordeaux, INSERM, Neurocentre Magendie, U1215, F-3300 Bordeaux, France; 7 The Francis Crick Institute, 1 Midland Road, London, NW1 1AT UK

**Keywords:** Neuroscience, Cell biology

## Abstract

Despite the central role of Rho GTPases in neuronal development, their functions in adult hippocampal neurogenesis remain poorly explored. Here, by using a retrovirus-based loss-of-function approach in vivo, we show that the atypical Rho GTPase Rnd2 is crucial for survival, positioning, somatodendritic morphogenesis, and functional maturation of adult-born dentate granule neurons. Interestingly, most of these functions are specific to granule neurons generated during adulthood since the deletion of *Rnd2* in neonatally-born granule neurons only affects dendritogenesis. In addition, suppression of *Rnd2* in adult-born dentate granule neurons increases anxiety-like behavior whereas its deletion in pups has no such effect, a finding supporting the adult neurogenesis hypothesis of anxiety disorders. Thus, our results are in line with the view that adult neurogenesis is not a simple continuation of earlier processes from development, and establish a causal relationship between Rnd2 expression and anxiety.

## Introduction

For decades, neurogenesis was believed to be restricted to embryonic and early postnatal periods in the mammalian brain. However, over the last 20 years, research has firmly established that new neurons are continuously born throughout the lifespan of mammals, especially in the hippocampal dentate gyrus (DG) [1]. Indeed, although the majority of dentate granule neurons (DGNs) are generated in early postnatal life, new DGNs continue to be produced throughout adulthood in mammals albeit at lower rates [2, 3], including in humans [4–7]. Mammalian adult hippocampal neurogenesis (AHN) is a highly regulated, activity-dependent process that is reported to be critically involved in hippocampus-dependent functions such as memory processing and mood regulation [8].

Comparable to embryonic neurodevelopment, AHN is a multi-step process that begins with a phase of proliferation of neuronal progenitors [9]. Once generated, immature neurons migrate from the subgranular zone (SGZ) of the DG to the inner granule cell layer (GCL) and differentiate into glutamatergic DGNs. Newborn neurons then extend dendrites toward the molecular layer, project axons through the hilus toward the CA3 [10], and finally integrate into the preexisting neural circuitry. However, only a few newborn cells are incorporated into the DG circuitry since the majority of these cells undergo apoptosis at the immature neuron stage [11, 12].

The finding that new neurons are generated throughout life has significant implications for brain repair. This has led to tremendous efforts to characterize how new neurons differentiate and integrate into adult neural circuitry. Yet, the cellular and molecular mechanisms that regulate the neurogenic process in the adult brain are not well characterized [13]. Furthermore, the mechanisms that differently regulate adult versus developmental neurogenesis are poorly understood. Thus, further studies are needed to provide new insights into the mechanisms involved in the generation, survival, and integration of individual newborn neurons in the adult versus developing brain. Such investigations might support the identification of new putative therapeutic targets for stimulating neurogenesis in health and disease.

Over the past several years, it has become clear that the Rho GTPases, the master regulators of the cytoskeleton, play a central role in various aspects of neuronal development, including migration, dendrite outgrowth, spine formation, and maintenance [14, 15]. Yet, their functions during adult neurogenesis remain largely unexplored [16]. In particular, in the context of AHN, only few studies have addressed the roles played by the most well-characterized members of this family, Cdc42, Rac1, and RhoA. Vadodaria et al (2013) showed that Cdc42 is involved in mouse neural stem/progenitor cell proliferation, dendritic development, and spine maturation, while Rac1 is important in the late steps of dendritic growth and spine maturation in adult hippocampal newborn neurons [17]. In the case of RhoA, pharmacological blockade of its signaling in vivo is reported to promote the survival of adult-born DGNs [18].

In this study, we have focused our attention on Rnd2 (also called Rho7/RhoN). Rnd2 belongs to the Rnd subfamily of atypical Rho proteins that lack intrinsic GTPase activity and are therefore constitutively bound to GTP [19]. Interestingly, among the 23 members of the Rho GTPase family [20], only *Rnd2* (and to a lesser extent *TC10*) is selectively enriched in the adult mouse SGZ and inner GCL, suggesting a potential prominent role in AHN [21]. In addition, Rnd2 regulates embryonic cortical neurogenesis especially radial migration through RhoA inhibition [22, 23], and was shown to control multiple aspects of neuronal development in vitro like neurite outgrowth and branching [24–26]. Thus, in view of these data, we postulated that Rnd2 might be a good candidate to play a key role in AHN. Here, to elucidate the in vivo function of Rnd2 in this process, we used a retrovirus-based loss-of-function approach. With this strategy, we demonstrate that Rnd2 is cell-autonomously required for the proper development of adult-born DGNs and critical for anxiety-like behavior. Furthermore, we find that retrovirally-transduced Cre-mediated loss of *Rnd2* expression in neonatally-born (P0) DGNs only impacts dendrite formation, suggesting that Rnd2 plays distinct roles during developmental and adult neurogenesis in the DG.

## Materials and methods

### Animals and genotyping

C57Bl6/J (Janvier) and *Rnd2*^*flox/flox*^ mice were housed, bred, and treated according to the European directive 2010/63/EU and French laws on animal experimentation. All procedures involving animal experimentation and experimental protocols were approved by the Animal Care Committee of Bordeaux (CEEA50) and the French Ministry of Higher Education, Research and Innovation (authorizations n°04997.02 and APAFIS#12546).

*Rnd2* conditional mutant allele is described in Supplementary information. *Rnd2* was targeted by homologous recombination to generate the *Rnd2*^*flox*^ allele. This allele contains one loxP site between the exon 1 and 2 and a second loxP site after the last exon. A neomycin (Neo) selection gene flanked by flippase recognition target (FRT) sites was inserted in 3ʹ of *Rnd2*. Following transmission of the mutation to the germline, the Neo gene was excised, giving rise to the *Rnd2*^*flox*^ allele. Upon Cre recombination, exons 2 to 5 of *Rnd2* are deleted.

Genotyping of *Rnd2* wild-type allele was performed with the following primers: forward, 5ʹ- CAGGGCACTTCTGATACAAAGC -3ʹ and reverse 5ʹ- TCTCACCCACCCCTGGCTGAT -3ʹ. Genotyping of *Rnd2* conditional mutant allele was performed with the same forward primer as for the wild-type allele (WT) and the following reverse primer 5ʹ- GTTTGTCCTCAACCGCGAGCTG -3ʹ. Mice were genotyped from genomic DNA purified from tail biopsies by PCR using these primers according to the following protocol. Tails were incubated overnight at 56 °C in Proteinase K (PK) buffer (100 mM Tris-HCl pH8, 5 mM EDTA, 0.2% SDS, 200 mM NaCl, 0.2 mg/mL PK). After centrifugation at 13200 rpm for 10 min, the supernatants were purified by vacuum on silica columns, according to the manufacturer’s protocol (Macherey-Nagel) and on Zephyr automatic station (Perkin-Elmer). PCR assay was carried out on a Bio-Rad C1000 thermal cycler, in a 20 µL volume, using GoTaq G2 Hot Start Green Master Mix (Promega), and 0.2 µM of common forward primer, 0.2 µM of flox Reverse primer, and 0.4 µM of WT reverse primer. PCR conditions were as follows: one cycle, 5 min at 95 °C; 37 cycles, 30 s at 95 °C, 30 s at 60 °C, 45 s at 72 °C; one cycle, 5 min at 72 °C. PCR products were analyzed on a Labchip GX microfluidic electrophoresis system (Perkin-Elmer) using the DNA5k kit.

### Constructs

The murine Moloney leukemia virus-based retroviral vectors CAG–GFP [27], CAG-GFP/Cre [12], CAG-RFP [28], and CAG-GFP-T2A-CreER^T2^ [29] were kind gifts from Dr. Fred Gage and the retroviral CAG-IRES-DsRed vector [30] from Dr Benedikt Berninger. Rnd2 and DNRhoA were cloned by PCR using pNeuroD1-Rnd2 [22] and pRK5-myc-RhoA-T19N (Addgene 12963) as templates respectively and then inserted into the SfiI/PmeI sites of the CAG-Neurog2-IRES-DsRed retroviral vector [30] to generate CAG-Rnd2-IRES-DsRed and CAG-DNRhoA-IRES-DsRed.

### Retrovirus production and injection into the mouse DG

High-titers of retroviruses were prepared as previously published [31] with a human 293-derived retroviral packaging cell line (293GPG) kindly provided by Dr. Dieter Chichung Lie. For experiments in adults, the retroviral solution (10^9^–10^10^ TU/ml) was injected into the DG of 12-week-old male *Rnd2*^*flox/flox*^ mice. Mice were anesthetized by intraperitoneal injection of ketamine (120 mg/kg; Imalgene® 1000, Merial)-xylazine (16 mg/kg; Rompun®, Bayer HealthCare) mixture and eye ointment was applied to prevent eyes from over-drying. A cranial subcutaneous injection of 0.1 μl of lidocaine (20 mg/ml; Lurocaïne®, Vetoquinol) and a dorsal subcutaneous injection of 0.1 μl of meloxicam (0.5 mg/ml; Metacam®, Boehringer Ingelheim) were performed before settling the mouse into the stereotaxic frame. Betadine was applied, then the skin was cut and a pulled microcapillary glass tube (1–5 μL, Sigma) was placed above bregma. Coordinates of the injection site from bregma were: anteroposterior: −2 mm, mediolateral: −1.8 mm, dorsoventral: −2.2 mm. A small hole was made on the skull using an electric drill, the microcapillary was loaded with the retroviral solution and introduced within the hole to reach the DG and stayed in place for one minute. Then 1.5 μl of retrovirus was injected at the rate of 0.5 μl every two minutes and the microcapillary was left again in the same position for two minutes after the end of infusion before being removed. Then the skin was stitched with absorbable sutures and the mouse was placed in a recovery chamber (37 °C) until it woke up.

For injections in pups, postnatal day 0 (P0) mice were anesthetized by hypothermia and 1 µl of retroviral solution (10^9^ TU/ml) was injected through the skin and skull into the lateral ventricle using pulled borosilicate needles and a Femtojet microinjector. To target the lateral ventricle, a virtual line connecting the right eye with the lambda was used and the needle was injected slightly caudal of the midpoint of this line as previously described [32]. Injected pups were placed in a recovery chamber at 37 °C for several minutes and then returned to their mother.

### Tamoxifen administration

For activation of the CreER^T2^ recombinase, animals were administered intraperitoneally with 150 mg/kg tamoxifen (Sigma, diluted in corn oil) for five consecutive days. Control animals were injected with the same volume of corn oil for five consecutive days.

### Tissue processing

Pups and adult mice were deeply anesthetized with an intraperitoneal injection of pentobarbital (100 mg/kg; Pentobarbital® sodique, Ceva), transcardially perfused with phosphate buffer saline (PBS, 0.1 M, pH = 7.3) and then with 4% paraformaldehyde (PFA) in PBS. Brains were dissected out of the skull and post-fixed overnight in 4% PFA, except for Rnd2 staining and Ankyrin-G immunostainings (2 h and 20 min post-fixation respectively). Brains were then cut coronally and serially (10 series of 40 µm sections) with a vibratome (Leica). Prior to sectioning, the brains of P3 pups were embedded in gels made with 3.5% agarose and 8% sucrose diluted in PB (0.1 M, pH = 7.3).

### Immunohistochemistry

For Rnd2 immunostaining, sections were washed three times with PBS for 5 min before blocking and permeabilization with PGT buffer (PBS, 2 g/L gelatin, 0.25% Triton X-100) as previously described (3 × 15 min) [33]. Sections were subsequently incubated overnight at 4 °C with a rabbit anti-Rnd2 antibody (1/500, Proteintech 13844-1-AP) diluted in PGT buffer. The following day, sections were washed 3 × 15 min with PGT and incubated with a biotinylated goat anti-rabbit secondary antibody (1/200, Vector Laboratories, BA-1000) in PGT for 2 h at room temperature (RT). After three washings of 15 min in PGT, sections were incubated with streptavidin Alexa Fluor® 488 (1/1000, Invitrogen) in PGT for 1 h at RT. Three final washes with PGT buffer were performed prior to a washing step in PBS and a final rinse in ultrapure water. Sections were mounted on glass slides with mounting solution (Aqua Polymount, Polysciences Inc.). For Rnd2/DCX co-immunostaining, a mouse anti-DCX (1/25, Santa Cruz, sc-271390) and a goat anti-mouse Alexa Fluor® 647 antibodies were used (1/1000, Jackson Immunoresearch).

For other immunostainings, sections were treated with PBS - 0.3% Triton X100 - 3% normal serum for 45 min after washings in PBS. They were then incubated overnight at 4 °C with the following primary antibodies diluted in PBS - 0.3% Triton X100 - 1% normal serum: mouse anti-Ankyrin-G (1/500, Millipore, MABN466), rabbit anti-cleaved caspase-3 (1/400, Cell Signaling, 9661), rabbit anti-DCX (1/2000, Sigma, D9818), chicken anti-GFP (1/1000; Abcam, ab13970), rabbit anti-Ki67 (1/1000; NovoCastra, NCL-Ki67-P), rabbit anti-DsRed (1/500, Clontech, 632496). Sections were then incubated for 2 h at RT with appropriate fluorescent secondary antibodies diluted in PBS––1% normal serum. TOTO-3 iodide (1/2000, Invitrogen) was added to the secondary antibody solution to label cell nuclei (Invitrogen). Images were acquired with a confocal microscope (Leica SP5).

### GCL volume measurement

The volume of the GCL was estimated as previously described [34] with some modifications. One of ten series of each brain was stained with DAPI. DAPI-stained sections were then acquired using a slide scanner Nanozoomer 2.0HT (Hamamatsu Photonics, France) and a TDI-3CCD camera. The area of the GCL on each section was measured using Image J. The volume of the GCL was estimated by multiplying the sum of the cross-sectional areas by the spacing, T, between sampled sections (T = 10 × 40 µm = 400 µm).

### Morphometric analysis

Dendrites and cell body of fluorescent dentate neurons were traced using a computer-controlled microscope-based system (Axio Imager A2 Zeiss microscope, 100X oil-immersion objective) with a software (Neurolucida® software; MicroBrigthField Bioscience) that provides neuron tracing tools to trace from a live camera image (QImaging).

For spine analysis, confocal stacks of images were obtained with a Leica SP5 confocal microscope (63X oil-immersion objective; XY dimensions: 41.0 µm; z-axis interval: 0.13 µm). The dendritic length of each segment was measured on Z projections, and the number of dendritic spines was counted using NeuronStudio software [35]. Before spine analysis, images were deconvoluted using AutoQuantX3 software (Media Cybernetics). A minimum of 30 dendritic segments per experimental group and time point were examined for spine analysis.

Morphometric analysis of the AIS was done as previously described [36] using a Leica SP5 microscope (63X water-immersion objective; XY dimensions: 82.0 µm; z-axis interval: 0.21 µm).

### RNA in situ hybridization

Brains, freshly perfused and post-fixed overnight with 4% PFA, were cut with a vibratome (40 µm). Nonradioactive RNA in situ hybridizations on floating brain sections were then immediately performed with digoxigenin-labeled riboprobes as previously described [37]. The riboprobes used to visualize the expression of *Rnd2* [22] and *Rnd3* [23] were previously described and the antisense RNA probe for *Rnd1* was prepared from IMAGE:3416797, GenBank accession number BE852181.

### Microdissection of the DG

**Fig. 1:**
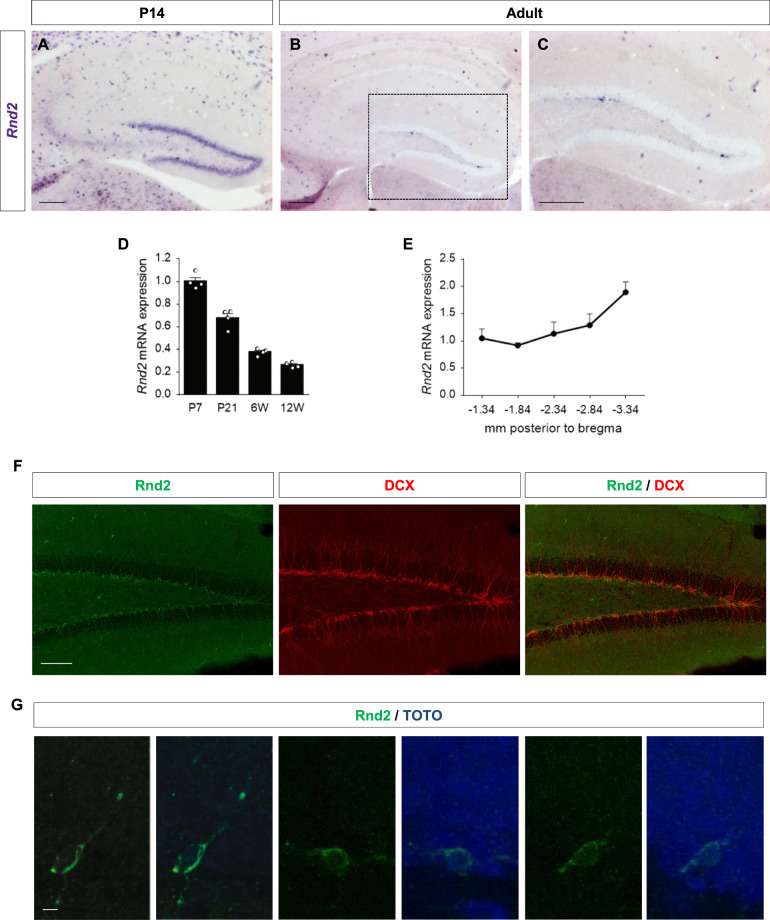
Rnd2 expression in the mouse DG. (**A**–**C**) Distribution of *Rnd2* transcripts in the hippocampus at postnatal day 14 (P14) (**A**) and at adult stage (12-week-old mouse) (**B**, **C**). The black rectangle in (**B**) shows the area enlarged in the inset C. **D** Analysis by real-time PCR of *Rnd2* mRNA expression in microdissected DG (SGZ and GCL) at different ages. Data are presented as fold change compared with the expression level at postnatal day 7 (P7) ± s.e.m (*n* = 4 mice per time point). P postnatal day, W weeks. **E** Analysis by real-time PCR of *Rnd2* mRNA expression along the septo-temporal axis in the adult (12-week-old) microdissected DG. Data are presented as fold change compared with the expression level at the anteroposterior coordinate −1.34 from the Bregma ± s.e.m (*n* = 5 mice per time point). **F** Immunostaining for Rnd2 and doublecortin (DCX) in the DG of a 12-week-old mouse (**G**) Rnd2 immunostained cells in the SGZ and inner GCL of the adult DG. Nuclei were labeled with TOTO. Scale bars represent 200 µm (**A**–**C**), 100 µm (**F**) and 5 µm (**G**). See also Supplementary Figs. 1–2.

Coronal sections (50 µm) were cut from frozen brains in isopentane using a cryostat (CM3050 S Leica) at −20 °C and mounted on polyethylene-naphthalate membrane 1 mm glass slides (P.A.L.M. Microlaser Technologies AG) that were pretreated to inactivate RNases. Sections were then immediately fixed for 30 sec with 95% ethanol and incubated with 75% ethanol for 30 s and with 50% ethanol for 30 s. Sections were stained with 1% cresyl violet in 50% ethanol for 30 sec and dehydrated in 50%, 75%, and 95% ethanol for 30 sec each, and finally, two incubations in 100% ethanol for 30 s were performed. Laser Pressure Catapulting (LPC) microdissection of the DG (SGZ and GCL) (Supplementary Fig. 1A) was performed using a PALM MicroBeam microdissection system version 4.6 equipped with the P.A.L.M. RoboSoftware (P.A.L.M. Microlaser Technologies AG). Laser power and duration were adjusted to optimize capture efficiency. Microdissection was performed at 5X magnification. Microdissected tissues were collected in adhesives caps and resuspended in 250 µl guanidine isothiocyanate-containing buffer (BL buffer from ReliaPrep™ RNA Cell Miniprep System, Promega) with 10 µl 1-Thioglycerol and stored at −80 °C until RNA extraction was done. For the analysis of *Rnd* expression at different time points (Fig. 1D and Supplementary information), the entire DG of each animal was microdissected. For the rostro-caudal analysis of *Rnd* expression (Fig. 1E and Supplementary information), DG in the right hemisphere from 10 following coronal sections of 50 µm were pooled for each coordinate. In both cases, we began to microdissect the DG from the section at the anteroposterior coordinate −1.34 mm from the Bregma (when the two blades of the DG were visible).

Total RNAs were extracted from microdissected tissues using the ReliaPrep™ RNA Cell Miniprep System (Promega) according to the manufacturer’s protocol. The integrity of the RNA was checked by capillary electrophoresis using the RNA 6000 Pico Labchip kit and the Bioanalyser 2100 (Agilent Technologies), and quantity was estimated using a Nanodrop 1000 (ThermoScientific). The RNA integrity numbers (RIN) were between 8.2 and 10.

### Microdissection of GFP+ cells

For this analysis, *Rnd2*^*flox/flox*^ mice were injected bilaterally with 2 µl of high-titer retroviral solution. Brains were dissected out of the skulls in PBS and incubated in 30% sucrose/PBS for three days at 4 °C under agitation. Brains were then frozen with isopentane and coronal sections (10 µm) were done using a cryostat and treated for microdissection as previously described but with some modifications. Sections were dehydrated in a series of pre-cooled ethanol baths (40 s in 95% and twice 40 s in 100%) and air-dried. Microdissection was performed at 63X magnification. Microdissected cells were collected in adhesives caps, resuspended in PK buffer (PK buffer from RNeasy FFPE Kit, Qiagen), and stored at −80 °C until extraction was done. Total RNAs were extracted using the RNeasy® FFPE Kit (Qiagen,) according to the manufacturer’s protocol. The RNA integrity numbers (RIN) were above 7/8. In the GFP group, a total of 6083 GFP+ cells was microdissected from 9 mice with a minimum of 561 cells and a maximum of 677 cells per animal. In the GFP/Cre group, a total of 5832 GFP+ cells was microdissected from eight mice with a minimum of 596 cells and a maximum of 878 cells per animal.

### Adherent cultures of adult hippocampal neural precursor cells

Adult hippocampal neural precursor cells were prepared from the DG of adult male *Rnd2*^*flox/flox*^ mice (eight-week-old) as described previously [38]. After enzymatic (Neural Tissue Dissociation Kit P, Miltenyi Biotec) and mechanical dissociation with fire-polished Pasteur pipettes, a step of enrichment was performed with Percoll (GE Healthcare) gradient centrifugation. Cells were then plated on poly-D-Lysine (10 µg/ml, Sigma) and laminin (10 µg/ml, Sigma) coated wells in initial proliferation medium: Neurobasal-A (Gibco), 2% B-27 (Gibco), 20 ng/ml epidermal growth factor (EGF, Peprotech), 20 ng/ml basic fibroblast growth factor (bFGF, Peprotech), 1% Glutamax (Gibco), 1% penicillin/streptomycin. Every two days, 50% of the medium was changed with fresh proliferation medium. Cells were passaged with accutase (StemCell Technologies) when cultures reached 80% confluence. For differentiation experiments, 2.10^4^ cells/cm [2] were initially plated in proliferation medium and after two days, differentiation was induced by replacing this medium with basic medium (Neurobasal, 2% B27, 1% Glutamax) supplemented with 5 ng/ml bFGF. After two days of differentiation and then every two other days, half of the medium was removed and replaced with basic medium without growth factors. For experiments with GFP or GFP/Cre retroviruses, cells were infected 4–6 h after plating (10^10^ TU/ml).

### Immunocytochemistry

For immunocytochemistry, cells were cultured on glass coverslips. After washing in PBS, cells were fixed 10 min at RT with PFA 4%. For Rnd2 staining, we used the PGT-based protocol described in “Immunohistochemistry section” with a fluorescent secondary antibody. For other stainings, cells were treated with PBS – 0.01% Triton X-100 – 1% bovine serum albumin (BSA, Sigma) for 30 min and incubated overnight at 4 °C with primary antibodies diluted in blocking solution: mouse anti-MAP2 (1/500, Sigma, M4403), chicken anti-nestin (1/400, Aves Labs, NES), mouse anti-Tuj1 (1/2000, Promega, G712A). Cells were then incubated with appropriate fluorescent secondary antibodies. Following this step, DAPI was added for 10 min. Images were acquired using an Eclipse Ti-U Nikon or a Leica SP5 microscope.

### Quantitative real-time PCR

RNA was processed and analyzed following an adaptation of published methods [39]. cDNA was synthesized from total RNA by using qSriptTM cDNA SuperMix (Quanta Biosciences). qPCR was performed using a LightCycler® 480 Real-Time PCR System (Roche). qPCR reactions were done in duplicate for each sample, using transcript-specific primers (Table 1), cDNA and LightCycler 480 SYBR Green I Master mix (Roche) in a final volume of 10 μl. PCR data were exported and analyzed in the GEASE software (Gene Expression Analysis Software Environment) developed in the Neurocentre Magendie (https://bioinfo.neurocentre-magendie.fr/outils/GEASE/). For the determination of the reference genes, the GeNorm method was used [39]. Relative expression analysis was corrected for PCR efficiency and normalized against two reference genes. The relative level of expression was calculated using the comparative (2^−∆∆CT^) method [40].

**Table 1:** Sequences of primers used for qPCR.

Gene	GenBank ID	Forward sequence (5ʹ-3ʹ)	Reverse sequence (5ʹ-3ʹ)
*Nestin*	NM_016701	GCTCTGGGCCAGCACTCTT	TCAGCCAGCCACACCTCTC
*Rnd1*	NM_172612	CCAGACGCTCCATCACTGAAA	GGGTTAGGCAAAAATGAGGGTT
*Rnd2*	NM_009708	CCTGTCACACATGAGCAGGGTA	GAGGAACATTCAACGTAGGACACA
*Rnd3*	NM_028810	GTTGATCGTTGCCAGAGTGATAAT	AAAGGAACTGCTCAAAAGGAGAAA
*Tuj1*	NM_023279	GATCCCCAACAACGTCAAGGTA	TGAAGGTGGATGACATTTTGAGC

### Western blotting

Cells were lysed in a buffer containing 50 mM HEPES pH 7.3, 0.15 mM EDTA pH 7.9, 4 mM EGTA, 150 mM NaCl and 1% Triton X-100 supplemented with a cocktail of protease inhibitors (aprotinin, leupeptin, pepstatin-A, and pefabloc, 10 μg/ml) for 30 min at 4 °C. After centrifugation for 15 min at 8000 rpm (4 °C), protein concentration was determined using a BCA kit (Thermo Scientific). Protein levels were normalized to 100 µg per sample and resuspended with 4× laemmli sample buffer (Bio-Rad) before boiling (5 min). Proteins were separated by SDS-PAGE using precast 4–20% gels (Bio-Rad) and then transferred onto a nitrocellulose membrane. Membranes were then blocked with Tris-Tween buffered solution (TTBS; 10 mM Tris, 200 mM NaCl, 0.05% Tween 20, pH 7.4) containing 5% non-fat dry milk for 1 h at RT and then incubated with primary antibodies (rabbit anti-Rnd2, 1/250, Proteintech, 13844–1-AP; mouse anti-beta actin, 1/2000, Sigma, A5316) overnight at 4 °C. HRP-labeled secondary antibodies (Jackson ImmunoResearch) were incubated the following day for 1 h at RT.

### Ex vivo electrophysiology

Recombinant retroviruses encoding for GFP or GFP/Cre were bilaterally injected into the DG of two respective groups of 12-week-old *Rnd2*^*flox/flox*^ male mice. Four weeks after retroviral injection, animals were sacrificed by dislocation. The brains were quickly removed and immersed in ice-cold oxygenated cutting solution containing in mM: 180 Sucrose, 26 NaHCO_3_, 12 MgCl_2_, 11 Glucose, 2.5 KCl, 1.25 NaH_2_PO_4_, 0.2 CaCl_2_, oxygenated with 95% O_2_/5% CO_2_ ~300 mOsm. Sagittal hippocampal slices (300 µm thick) were obtained using a vibratome (VT1200S, Leica) and transferred for 30 min into a 34 °C bath of oxygenated ACSF containing in mM: 123 NaCl, 26 NaHCO_3_, 11 Glucose, 2.5 KCl, 2.5 CaCl_2_, 1.3 MgCl_2_, 1.25 NaH_2_PO_4_ ~305 mOsm. After a minimum of 60 min recovery at RT (22-25 °C), slices were transferred to a recording chamber in ACSF at 32 °C. Recordings were performed using a Multiclamp 700B amplifier (Molecular devices) in fluorescent granule neurons clamped with glass pipettes (3-5 MΩ) filled with an internal solution containing in mM: 135 K-Gluconate, 10 KCl, 10 HEPES, 1 EGTA, 2 MgCl_2_, 0.3 CaCl_2_, 7 Phosphocreatin, 3 Mg-ATP, 0.3 Na-GTP; biocytin 0.4%; pH = 7.2; 290 mOsm. These cells were identified by their GFP expression, their morphology, and soma location in the SGZ/GCL using a contrast microscope (Axio Examiner.A1, Zeiss) equipped with a fluorescent light (Colibri controller, Zeiss). Neuronal excitability was measured using 500 ms steps of current injections from -150 to 450 pA with increasing steps of 10 pA. Immunostaining of both GFP and biocytin was performed to confirm the identity of the recorded cells.

### Behavior

For experiments with adults, 12-week-old male *Rnd2*^*flox/flox*^ mice were bilaterally injected with 1.5 µl of GFP or GFP/Cre retroviruses. Two batches of animals were used. The number of animals in each group was as follows: Batch 1 (GFP, 13 mice; GFP/Cre, 14 mice), Batch 2 (GFP, 11 mice; GFP/Cre, nine mice). For the first batch, the titers of GFP and GFP/Cre retroviruses were 3–4.10^9^ TU/ml and for the second batch, they were estimated at 5–6.10^10^ TU/ml.

For experiments with pups, P0 *Rnd2*^*flox/flox*^ mice were bilaterally injected with 1 µl of GFP or GFP/Cre retroviruses. The titers of GFP and GFP/Cre retroviruses were 1.10^9^ TU/ml. Nine GFP and 8 GFP/Cre mice were used for the analysis.

The behavioral sequences are presented in Supplementary information. For all behavioral tests, animals were placed in the test room 30 min before the beginning of the experiment. For all procedures, experimenters were blind to the virus injected.

### Open-field test (OF)

Mice were placed in one corner of a square open-field (50 × 50 cm, 200 lux). Exploratory behavior was monitored for 10 min. Time spent in the center (35 × 35 cm) and the distance ratio (distance traveled in the periphery divided by the total traveled distance) were automatically measured by a video-tracking system connected to a camera (Videotrack, ViewPoint). These two parameters were used for z-open field score calculation.

### Emergence test

The emergence test was done in the same open-field but with brighter light (~300 lux). Mice were placed in a dark cylinder (10 × 6.5 cm, grey PVC) in one corner of the arena. The behavior was monitored for 5 min using the same system as in the open-field test (Videotrack, ViewPoint). Latency to emerge from the cylinder and the number of re-entries in the cylinder were automatically measured and used for z-emergence score calculation.

### Elevated Plus Maze (EPM)

Behavior in the EPM was measured using a cross maze with two open and two closed arms (37 × 6 cm arms, 75 lux) placed 115 cm above the ground. Mice were placed at the center of the cross, facing one of the open arms and their behavior was monitored for 5 min. Time spent in open arms as well as the ratio between entries in open arms divided by entries in closed and open arms were measured using the video-tracking system (Videotrack, ViewPoint) and used for z-EPM score calculation.

### Light/Dark

The apparatus used for this test was composed of a strongly illuminated chamber (36 × 36 cm, ~350 lux) and a dark chamber (36×23 cm, ~10 lux) separated by a wall with a door (10 × 10 cm) allowing animals to travel freely between the two compartments. Mice were placed in the lit chamber and left exploring the apparatus for 5 min. Latency to leave the lit chamber and time spent in it were manually measured and used for z-light dark score calculation.

### Sucrose preference

In the sucrose preference test, mice were exposed in their home cage to two drinking bottles for 72 h. During the first 24 h, habituation was performed with the two bottles filled with water. For the test phase, one of the bottles was filled with 30 mL of 4% sucrose water while the second bottle was filled with water. Bottles were placed side by side for 24 h, then for the last 24 h positions were reversed. Sucrose preference ratio was calculated as follows and used for z-sucrose preference score calculation:

$$Sucrose\,preference = \frac{{{{{{{\mathrm{{\Delta}}}}}weight}}_{sucrose}}}{{{{{{{\mathrm{{\Delta}}}}}weight}}_{sucrose} + {{{{{\mathrm{{\Delta}}}}}weight}}_{water}}}$$

### Forced swim test (FST)

The FST device consists of a glass cylinder 16 cm in diameter and 25 cm high, filled with water to a height of 15 cm and placed in a strongly illuminated room (~350 lux). Water was maintained at ~26 °C. Mice were placed in the apparatus and behavior was monitored for 6 min. Latency to immobility and total immobility time in the last 4 min of the test were measured and used for z-FST score calculation.

### Morris Water Maze (MWM)

The apparatus was a white circular swimming pool (150 cm in diameter and 60 cm deep) located in a room with various distal visual cues (80 lux). The pool was filled with water maintained at 19 °C and made opaque by the addition of a non-toxic white cosmetic adjuvant. The escape platform (14 cm diameter) was hidden underwater so that its top surface was 0.5 cm below the surface of the water. In this task, mice were required to locate the hidden platform using distal cues and mice behavior was monitored using a camera and a video-tracking system (Videotrack, ViewPoint). For the entire training period, the platform stayed in the same position, and mice were tested with variable random start positions (see Supplementary information). Mice were trained in 3 trials a day, each trial being separated by a 5 min interval. A trial was terminated when the animal climbed onto the platform. Mice that failed to find the platform within the 60 s cutoff time were placed onto the platform by the experimenter and had to stay there for 15 s before being placed back in their home cage. The releasing point (starting point) differed for each trial and different sequences of releasing points were used day to day. Three days after the last training trial, the hidden platform was removed and the memory for the platform location was assessed during a probe test (see Supplementary information). During this test, mice were allowed to freely swim in the water maze for 60 s and performances were assessed by time spent in the target quadrant where the platform was located.

### Contextual fear conditioning and discrimination

Mice were exposed to contextual fear conditioning in order to test their ability to discriminate similar contexts. Conditioning was done in a transparent plexiglass cage (26 x 25 x 17 cm) allowing access to visual cues of the environment with a floor composed of 42 stainless steel rods, separated by 3 mm, which were wired to a shock generator and scrambler (Imetronic). In context A, the cage was illuminated (90 lux), the testing room was strongly illuminated (250 lux) and visual cues were placed on the walls of the room. The cage was cleaned between each mouse with a 70% ethanol solution and the experimenter wore latex gloves. For context B, the testing room was lit with a low light (50 lux) and the cage was not illuminated. Dark panels were placed proximal to the cage, on which visual cues different from those used in context A were placed. Furthermore, a plastic boat containing used litter was added under the steel bars of the cage, and lemon scent was added in the cage. Between each mouse, the cage was cleaned with 30% acetic acid solution and the experimenter wore nitrile gloves.

For the conditioning phase, mice were individually transported from the resting room to the experimental room and placed in the context A conditioning chamber. After 180, 240, and 300 s, they received a single footshock (0,7 mA, 1 s) and remained in the chamber for 1 min (see Supplementary information) after the last shock before being transported back to their housing room. On the following day mice were tested for context discrimination. In this goal, mice were again individually transported to the experimental room and placed in the context A conditioning chamber for 5 min without delivery of a footshock. Three hours later, mice were then exposed to the context B conditioning chamber for 5 min, again without any footshock (see Supplementary information). During the conditioning phase and the testing phase, mice behavior was monitored with a video-tracking system (Freezing, Imétronic). Freezing behavior, defined as behavioral immobility except for breathing movements, was measured. Discrimination of contexts was evaluated comparing the immobility time in each context, and a discrimination ratio was calculated as follows:

$$Discrimination\,ratio = \frac{{immobility\,time_{context\,A}}}{{immobility\,time_{context\,A + context\,B}}}$$

### Criteria of exclusion

Most tests used in this study rely on exploration and locomotor activity, therefore total distance traveled in these tests was used as an index of locomotor activity. Although no animal was excluded on the basis of the total traveled distance, several other criteria were determined to exclude mice that did not follow the rules of the designed tests. For example mice that did not swim and instantly started floating during the MWM and the FST would be excluded for the final analysis of performances, as well as mice that did not leave the cylinder in the emergence test. In the EPM, mice that stayed 240 s in the center or less than 1 s in open or closed arms were excluded from analysis, as well as mice that explored less than two different arms. For behavioral experiments with adults, no animal was removed from the analysis after euthanasia because all of them showed a significant number of GFP+ cells on both side. For behavioral experiments with pups, only animals with GFP+ cells on both sides were kept for the analysis.

### Z-score calculation

Z-scores are mean-normalization of the results and allow for comparison of related data across this study. They indicate how many standard deviations (σ) an observation (X) is above or below the mean of a control group (µ) and were calculated as previously described [41]:

$$Z = \frac{{X - {{{{{\mathrm{\mu }}}}}}}}{{\upsigma }}$$

Z-score values were calculated for test parameters measuring emotionality. The directionality of scores was adjusted so that increased score values reflect increased dimensionality (anxiety or depression-related behavior). For instance, decreased time spent in open arms in the EPM or decreased time spent in the center of the open-field test, compared to control, were converted into positive standard deviation changes compared to group means indicating increased anxiety-related behavior. On the other hand, increased immobility time in the FST for example, is directly related to increased depression-like behavior and was not converted.

As an example, z-score in the open-field (Z_OF_) was calculated for each animal, using normalization of “time in the center” (TC) and “distance in periphery/total distance ratio” (DR) values.

$$Z_{OF}=\frac{\left(\frac{X-\mu}{\sigma}\right)TC+\left(\frac{X-\mu}{\sigma}\right)DR}{Number\, of\, parameters}$$

Similarly, z-scores were calculated for the emergence test (Z_emergence_), the EPM (Z_EPM_), and the light/dark test (Z_L/D_). z values obtained for each test were then averaged to obtain a single z-anxiety score:

$$Anxiety\,score = \frac{{Z_{OF} + Z_{emergence} + Z_{EPM\,4w} + Z_{EPM\,10w}}}{{Number\,of\,tests}}$$

The same calculations were done with the z-scores of the sucrose preference test (Z_SP_) and the FST (Z_FST_), and an individual “depression score” was calculated as for the z-anxiety score.

### Statistical analysis

Statistical analyses were performed using the GraphPad Prism software. No statistical methods were used to predetermine sample sizes, but our sample sizes are similar to those generally employed in the field. All mice were assigned to different experimental conditions randomly. Investigators were blind to group allocation in behavioral experiments. Results are presented as mean ± s.e.m. (standard error of the mean). The statistical test used for each experiment, and sample size (*n*) are indicated in the corresponding figure legend or in the Methods. Statistical values are mentioned in the text.

## Results

### Rnd2 is enriched in the temporal SGZ of the adult mouse DG

We began this study by examining the expression of Rnd2 in the mouse DG. We first observed by RNA in situ hybridization and real-time PCR that *Rnd2* expression decreases significantly in the DG between the postnatal and the adult period (Fig. 1A–D), similarly to related family members *Rnd1* and *Rnd3* (Supplementary Fig. 1A, B). However, in the adult brain, *Rnd2* shows a different pattern of expression compared to other members of the Rnd family, particularly interesting in the context of adult neurogenesis. Indeed, *Rnd2* is restricted to the SGZ and inner GCL of the DG (Fig. 1B, C), whereas *Rnd1* and *Rnd3* are more prominently localized to the CA1-CA3 hippocampal fields and in the DG (Supplementary Fig. 1C). In addition, whereas *Rnd1* and *Rnd3* levels do not fluctuate dramatically along the septo-temporal axis (Supplementary Fig. 1D), *Rnd2* mRNA expression increases in the temporal part of the adult DG (Fig. 1E; F_4,16_ = 7.16, *p* = 0.002). This result is consistent with a RNA-seq study (http://hipposeq.janelia.org), which reported a significantly higher level of *Rnd2* mRNA expression in the temporal DGNs relative to septal DGNs [42]. By immunohistochemistry, we confirmed, at the protein level, the enrichment of Rnd2 in the SGZ and inner GCL of the adult DG, where doublecortin (DCX) positive cells are located (Fig. 1F). To determine when Rnd2 is expressed during the maturation of newborn neurons in the DG, we made use of published single-cell RNA-seq data prepared from young mouse DG (http://linnarssonlab.org/dentate/) [43]. Interestingly, these data, in accordance with the morphology of Rnd2+ cells in the DG (Fig. 1G), reveal that *Rnd2* is particularly enriched in neuroblasts and to lesser extent in immature granule neurons (Supplementary Fig. 2) Altogether these results indicate that Rnd2 is particularly enriched in newborn neurons of the adult DG, suggesting that this atypical Rho GTPase might be involved in AHN.

### Rnd2 is critical for the survival of adult-born DGNs

To address the role of Rnd2 in AHN, we deleted *Rnd2* specifically in adult-born DGNs using a loss of function approach based on retrovirus-mediated single-cell gene knockout in new neurons in vivo [44]. The principle of the technique is to deliver the Cre recombinase selectively to newborn neurons, using a retroviral vector, into the DG of *Rnd2*^*flox/flox*^ adult mice (Supplementary Fig. 3A). By injecting retroviruses expressing Cre fused to green fluorescent protein (GFP/Cre, *Rnd2* deletion), or GFP only in control condition, into the DG of adult *Rnd2*^*flox/flox*^ mice (12-week-old), we first confirmed, after microdissection of GFP+ cells followed by RT-PCR, that *Rnd2* mRNA expression was strongly decreased in the GFP/Cre group compared to control whereas *Rnd1* and *Rnd3* mRNA expressions were not changed (Supplementary Fig. 3B; t_15_ = 4.42, *p* < 0.001 for Rnd2 analysis). Indeed, seven days post injection (dpi), the expression of *Rnd2* was already decreased by almost 80% (Supplementary Fig. 3B). The efficient depletion of Rnd2 was also confirmed at the protein level, in vitro, using cultures of adult neural precursor cells prepared from the DG of adult *Rnd2*^*flox/flox*^ mice and infected with a retrovirus expressing GFP/Cre or GFP (Supplementary Fig. 4).

**Fig. 2:**
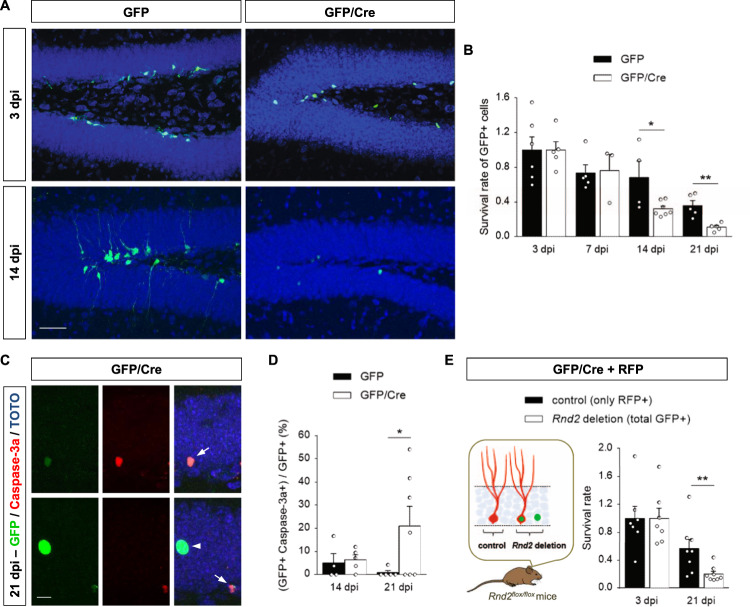
The absence of *Rnd2* reduces the survival of adult-born DGNs. **A** Images of the DG illustrating the reduction of GFP+ cell number after *Rnd2* deletion (GFP/Cre panel) compared to control (GFP panel) at 14 days post injection (dpi). No difference was observed at three dpi. Nuclei were labeled with TOTO. **B** Survival rate of GFP+ cells at different time points. Mean ± s.e.m.; unpaired two-tailed Student’s *t*-test; *p < 0.05, **p < 0.01 (*n* = 3–7 mice). **C** Immunostaining for GFP and activated caspase-3 (caspase-3a) in the DG, 21 days after GFP/Cre virus injection. TOTO labels nuclei and identifies the GCL. Arrowhead shows a non apoptotic cell and arrows indicate apoptotic cells. **D** Quantification of the percentage of transduced cells that are caspase-3a+ at 14 and 21 dpi. Mean ± s.e.m.; unpaired one-tailed Student’s *t*-test; *p < 0.05 (*n* = 4–7 mice). **E** The retroviral co-injection strategy allows to analyze in the same mice *Rnd2*-deleted and control new neurons. The graph shows the survival rate of control (RFP+ only) and *Rnd2*-knockout (GFP+ only and GFP+/RFP+) new neurons in *Rnd2*^*flox/flox*^ mice. Mean ± s.e.m.; paired two-tailed Student’s *t*-test; **p < 0.01 (*n* = 7–8 mice). Scale bars represent 50 µm (**A**) and 10 µm (**C**). See also Supplementary Figs. 3–6.

Once validated, we used this viral strategy to study the role of Rnd2 in the development of adult-born DGNs. We first showed that *Rnd2* deletion in these cells does not affect neuronal progenitor proliferation and neuronal differentiation (Supplementary Fig. 5). Intriguingly, we noticed that the number of GFP+ labeled cells in *Rnd2*-deleted animals was consistently lower compared to control animals 2–3 weeks after retroviral injection (Fig. 2A). In order to understand if Rnd2 expression is relevant to the survival of adult hippocampal newborn neurons, we quantified the number of GFP+ cells in the DG of *Rnd2*^*flox/flox*^ mice, injected with Cre/GFP or GFP expressing retrovirus, across multiple time points post-injection (Fig. 2B). Importantly, for each type of virus, the same preparation was used for all time point analyzed. In control condition, the number of retrovirus-labeled GFP cells at 21 dpi was reduced by 64% compared to three dpi (Fig. 2B), a finding which is consistent with previous studies [11, 12]. Consistent with our first observations, the suppression of *Rnd2* substantially decreased the number of newborn DGNs from 14 dpi and, at 21 dpi, only 10.9% ± 1.9% of *Rnd2*-deleted cells survived compared to 35.7 ± 5.7% of control cells (Fig. 2A, B; t_9_ = 2.54, *p* = 0.03 at 14 dpi; t_8_ = 4.12, *p* = 0.003 at 21 dpi).

To determine whether the decrease in the number of new neurons after *Rnd2* suppression was due to their elimination through apoptosis, we performed immunostaining for activated caspase-3 (caspase-3a, Fig. 2C). Since apoptotic cells are rapidly cleared [45, 46], the probability of detecting apoptotic newborn cells is low. This is reflected in our observation that only a small fraction of GFP+ cells were immunopositive for caspase-3a in the control group at 14 and 21 dpi (Fig. 2D). Nonetheless, the proportion of transduced cells expressing caspase-3a was significantly increased at 21 dpi in the GFP/Cre condition compared to GFP (Fig. 2D; t_10_ = 1.98, *p* = 0.04) indicating that *Rnd2* suppression in newborn neurons leads to enhanced programmed cell death.

To further confirm these results, we used a retroviral co-injection strategy [12] in which we injected *Rnd2*^*flox/flox*^ mice with a retrovirus expressing GFP/Cre together with a red fluorescent protein (RFP) expressing control virus. This approach enables us to investigate the viability of *Rnd2*-knockout (GFP+/RFP+ cells and GFP+ only) and control new neurons (RFP+ only) in the same mice (Fig. 2E). Consistent with our previous results, we found that the number of *Rnd2*-deleted cells was significantly lower at 21 dpi compared to control cells (Fig. 2E; *t*_7_ = 3.71, *p* = 0.008). Importantly, when we performed the same analysis in wild-type C57Bl6/J mice (Supplementary Fig. 6A), there was no difference between the two categories of cells, thus excluding the possibility that the observed effect is due to Cre toxicity. Furthermore, we validated that the exacerbated cell death is specific to *Rnd2* suppression since the survival of *Rnd2*-knockout cells could be restored to levels that are not significantly different from control by co-delivery of a retroviral vector encoding Rnd2 expression construct (Supplementary Fig. 6B, C). Thus, suppression of *Rnd2* in adult-born DGNs impairs their survival.

Lastly, since pharmacological inhibition of RhoA signaling was shown to enhance the survival of adult-born DGNs [18] and because Rnd2 inhibits RhoA in migrating cortical neurons [23], we tested whether inhibition of RhoA could counteract the death induced by the loss of *Rnd2* in adult-born DGNs. We found that the loss of *Rnd2-*knockout cells could not be restored by co-delivery of dominant-negative (DN)-RhoA (Supplementary Fig. 6B, C), suggesting that the action of Rnd2 on adult newborn survival does not involve RhoA inhibition.

### Rnd2 is required for the correct positioning, morphogenesis, and functional maturation of adult-born DGNs

**Fig. 3:**
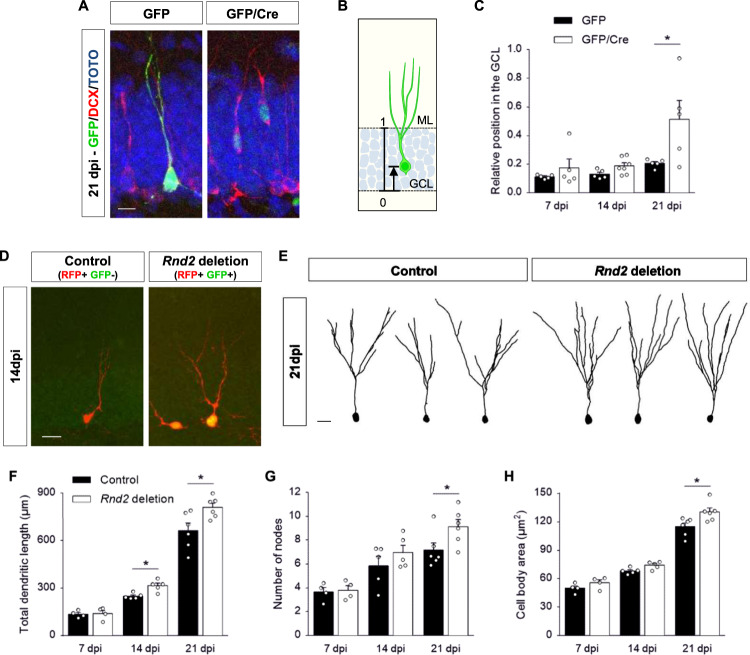
*Rnd2* deletion affects the positioning and the morphogenesis of adult-born DGNs. **A** Representative images of adult newborn neurons transduced with GFP or GFP/Cre retrovirus at 21 dpi in the DG. Doublecortin (DCX) identifies immature neurons and TOTO-3 labels nuclei. **B** Schematic depiction of the analysis of the relative positions of newborn neurons in the granular cell layer (GCL) of the DG. ML, molecular layer. **C** Relative positions of newborn neurons within the GCL in control group (GFP) and after *Rnd2* deletion (GFP/Cre). Mean ± s.e.m.; unpaired two-tailed Student’s *t*-test; *p < 0.05 (*n* = 5–7 mice, a minimum of six cells were analyzed per animal). **D** Representative images of control (RFP+ GFP−) and *Rnd2*-depleted (RFP+ GFP+) newborn neurons in the DG of *Rnd2*^*flox/flox*^ mice, 14 days after the injection of a mixture of GFP/Cre and RFP retroviruses. **E** Representative tracings of control and *Rnd2*-deleted newborn neurons in the adult DG at 21 dpi. **F**–**H** Quantification of the total dendritic length (**F**), the number of nodes (**G**) and the cell body area. Mean ± s.e.m.; paired two-tailed Student’s *t*-test; *p < 0.05, (*n* = 4–6 mice, a minimum of 4 cells were analyzed per animal). Scale bars represent 10 µm (**A**) and 20 µm (**D**, **E**). See also Supplementary Fig. 7.

We next examined the role of Rnd2 in the migration of adult-born DGNs. Previous studies have established that adult-born DGNs migrate into the GCL during the second week after birth [47] and they contribute mostly to the inner third of the GCL and to a lesser extent to the mid third [11, 48]. To analyze the impact of *Rnd2* deletion on this developmental step, we determined the relative position of GFP and GFP/Cre retrovirus-labeled cells expressing DCX in the GCL at 7, 14, and 21 dpi (Fig. 3A). This relative position was examined as already described [31]: the inner border of the GCL was defined as the baseline to measure cell migration and the perpendicular distance from this baseline to the center of each cell body was measured and normalized by the thickness of the GCL (Fig. 3B). At 21 dpi, we found that *Rnd2*-knockout neurons migrated a further distance in the GCL when compared to control neurons (Fig. 3C; *t*_8_ = 2.34, *p* = 0.05), indicating that Rnd2 influences the positioning of newborn neurons in the GCL of the adult DG.

While migrating, adult-born DGNs develop their dendritic arborization [47]. Three weeks after birth, their overall morphology resembles that of neurons at later time points [27]. To determine whether Rnd2 is also important for this process, we examined the morphology of newborn neurons in *Rnd2*^*flox/flox*^ brains injected with a mixture of GFP/Cre and RFP retroviruses. In the same animals, we were able to reconstruct the somatodendritic structure of control cells (cells expressing only RFP) and *Rnd2*-deficient cells (cells expressing GFP and RFP) (Fig. 3D). Interestingly, our results showed that *Rnd2*-deleted neurons have longer (Fig. 3E, F; *t*_4_ = 3.64, *p* = 0.02 at 14 dpi; *t*_5_ = 3.36, *p* = 0.02 at 21 dpi) and more branched dendrites compared to control neurons (Fig. 3E, G; *t*_5_ = 3.39, *p* = 0.02 at 21 dpi). Furthermore, we found that cell bodies of *Rnd2*-deleted neurons were significantly larger than those of control neurons at 21 dpi (Fig. 3H; *t*_5_ = 3.17, *p* = 0.02). To note, a similar control experiment was performed in WT mice and show that the flanking of *Rnd2* by loxP sites does not cause any somatodendritic modification (Supplementary Fig. 6D–F). We then analyzed the impact of *Rnd2* deletion on spine density and morphology at 21 and 28 dpi, a stage representing the peak of spine growth [27]. We observed that the total spine density of *Rnd2*-deficient newborn neurons was not statistically different compared to controls and no major difference in spine morphology was found between the two groups (Supplementary Fig. 7A–D). At the axon level, we did not observe any modification of the axon initial segment (AIS) length, thickness, and distance from the soma after *Rnd2* deletion (Supplementary Fig. 7E–H). Altogether, these results show that besides their correct distribution in the GCL, Rnd2 controls the morphogenesis of adult-born DGNs, including soma size and dendritic tree extent.

**Fig. 4:**
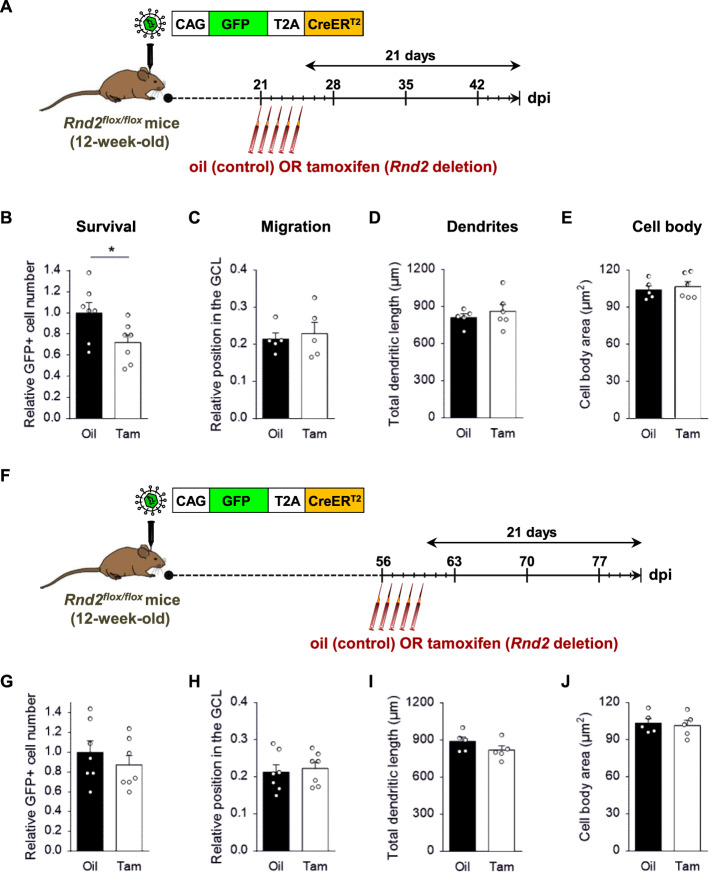
*Rnd2* deletion impacts directly the survival of adult-born DGNs during a specific period of their development. **A**, **F** Experimental designs. A retrovirus expressing GFP together with a conditionally active form of Cre recombinase, which is activated upon tamoxifen, was injected into the DG of adult *Rnd2*^*flox/flox*^ mice. Three (**A**) or eight weeks (**F**) after virus injection, tamoxifen (150 mg/kg, daily for five days), or oil in control group, was injected, and animals were sacrificed 21 days after the last injection of tamoxifen. dpi days post injection. **B**–**E**, **G**–**J** The relative number of GFP+ cells, the relative position in the GCL, and the morphology of transduced cells were quantified 21 days after the last injection of tamoxifen. Mean ± s.e.m., Unpaired two-tailed Student’s *t*-test; *p < 0.05 (*n* = 7 animals per group). See also Supplementary Fig.  8.

The aforementioned results then raised the following question: do *Rnd2*-deleted neurons die because of their mispositioning and/or aberrant morphology or because Rnd2 has a direct effect on the survival of adult newborn neurons? To discriminate between these two scenarios, we delivered a retrovirus expressing an inducible Cre [29] in order to delete *Rnd2* expression at a specific stage, in particular 3 weeks after birth, when new neurons have reached their final position and established their arborization (Fig. 4A). Using this approach, we found that *Rnd2* deletion led to a significant decrease in the survival of adult-born DGNs (Fig. 4B; *t*_12_ = 2.31, *p* = 0.04), whereas their positioning and morphology were not perturbed (Fig. 4C–E), indicating that Rnd2 controls the survival of these cells independently of their position and morphology. Interestingly, while induction of *Rnd2* deletion at 28 dpi still led to a decrease of newborn neuron survival (Supplementary Fig. 8; *t*_11_ = 2.75, *p* = 0.02 for survival), *Rnd2* deletion at 56 dpi did not (Fig. 4F–J; *t*_12_ = 0.84, *p* = 0.42 for survival). Therefore, these experiments suggest that Rnd2 is critical for adult hippocampal newborn neuron survival during a defined period of their development, at the immature stage.

**Fig. 5:**
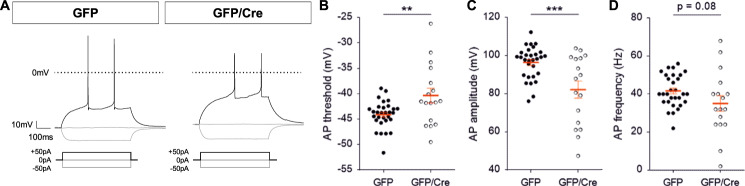
*Rnd2* deletion affects the intrinsic excitability of adult-born DGNs. **A** Representative spiking pattern of control (GFP) and *Rnd2*-deleted (GFP/Cre) newborn neurons in response to current injection (0 or ± 50 pA for 500 ms). **B**–**D** Quantification of the threshold of the first elicited AP (**B**), the maximum amplitude of this first AP (**C**) and the maximum AP frequency elicited by current injection (**D**). Mean ± s.e.m; unpaired two-tailed Student’s *t*-test; **p < 0.01, ***p < 0.001 (*n* = 30 neurons from 4 mice in GFP group and *n* = 17 neurons from eight mice in GFP/Cre group). See also Supplementary Fig. 9.

To further understand the impact of Cre-mediated *Rnd2* deletion in adult-born DGNs, we performed electrophysiological recordings of adult-born DGNs in acute slices from GFP or GFP/Cre virus-infused animals under whole-cell current-clamp configuration. Through this approach, we found that the membrane properties of four-week-old adult-born DGNs (membrane resistance, capacitance, and resting potential) were not affected by the suppression of *Rnd2* (Supplementary Fig. 9A–D). In response to a depolarizing current step, we also examined the ability of new neurons to generate action potentials (APs), a hallmark of neuronal maturation. As compared to control, we found that the AP threshold was significantly increased in *Rnd2*-deleted neurons (Fig. 5A, B; −40.4 ± 1.4 mV versus −44.1 ± 0.5 mV; *t*_45_ = 2.98, *p* = 0.005). Moreover, the AP amplitude was smaller in *Rnd2*-deficient newborn neurons (Fig. 5A, C; 82.1 ± 4.5 mV versus 96.4 ± 1.5 mV; *t*_45_ = 3.64, *p* = 0.0007) and the maximal frequency of these neurons to fire APs tended to be reduced compared to control neurons (Fig. 5D; 35.1 ± 4.1 Hz versus 41.6 ± 1.5 Hz; *t*_45_ = 1.78, *p* = 0.08), which altogether indicates that *Rnd2*-deleted newborn neurons have a decreased ability to fire APs. Since AIS morphometric parameters were not modified (Supplementary Fig. 7E–H), this impaired excitability might be caused by the abnormal morphology of *Rnd2*-deleted neurons [49, 50]. These results suggest that Rnd2 is critical for the proper development of intrinsic excitability of adult-born DGNs and consequently for their integration into the hippocampal circuitry.

### Rnd2 expression in adult-born DGNs controls anxiety-like behavior

It has been reported that new neurons in the adult DG are of pivotal importance for several hippocampal-dependent functions [13] including spatial navigation [51] as well as behavioral pattern separation [52–54], a process allowing to distinguish highly similar events, objects or contexts. In addition, AHN has been shown to be implicated in the regulation of affective states [8], particularly in anxiety [55]. Given our observation that Rnd2 is crucial for the survival of adult newborn neurons and their maturation within the DG, we studied whether the deletion of *Rnd2* specifically in these cells impairs hippocampal-dependent memory, anxiety, and/or depression-like behaviors. In this goal, two batches of adult *Rnd2*^*flox/flox*^ mice were injected bilaterally into the DG with high-titer retroviruses expressing GFP or Cre/GFP and behaviorally tested at least four weeks after the injection (Supplementary Fig. 10A).

To evaluate memory in these mice, we first studied spatial navigation according to a classical procedure in the Morris water maze (MWM) that allows to test reference memory (Supplementary Fig. 10B). We found that spatial memory was intact following *Rnd2* deletion in adult-born DGNs (Supplementary Fig. 10C). Moreover, no memory deficit was observed during the probe test, performed three days after the completion of the learning phase, indicating that memory retention and recall were intact (Supplementary Fig. 10D). We then tested our animals for their ability to discriminate similar contexts (Supplementary Fig. 10E) in a contextual fear discrimination task. This ability is sensitive to disruption of adult neurogenesis [54] and is thought to be facilitated by pattern separation, a neural computation process through which two similar input patterns are separated from each other via an orthogonalization of sensory input information [56]. Results show that Cre-mediated deletion of *Rnd2* in adult-born DGNs did not impact contextual memory (Supplementary Fig. 10F) and did not alter the ability of mice to discriminate the conditioning context (A) from a highly similar one (B) since both groups showed a lower freezing response in context B compared to context A, 24 h or five weeks after conditioning (Supplementary Fig. 10F, G). This result suggests that in our experimental conditions, Cre-mediated deletion of *Rnd2* in adult-born DGNs does not affect behavioral pattern separation.

**Fig. 6:**
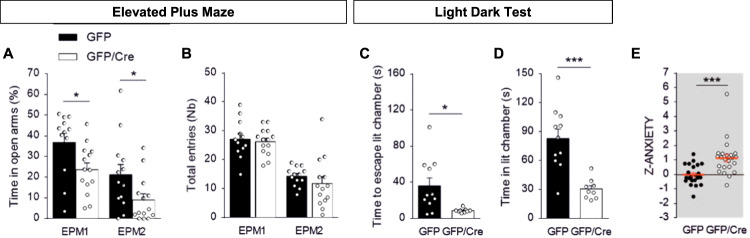
*Rnd2* deletion in adult-born DGNs increases anxiety-like behavior. **A** Time spent in the open arms (in % of time spent in the open arms and in the closed arms) and (**B**) number of total entries in the elevated plus maze (EPM), four (EPM1) or 10 weeks (EPM2) after GFP or GFP/Cre retrovirus injection. Mean ± s.e.m., Unpaired two-tailed Student’s *t*-test; *p < 0.05. **C** Time to escape and (**D**) time spent in the lit area in the light/dark test, five weeks after retroviral injection. Mean ± s.e.m., Unpaired two-tailed Student’s *t*-test; *p < 0.05, ***p < 0.001. **E** z-anxiety score calculated by averaging z-score values of the different tests used to assess anxiety-like behavior. An increased score value reflects increased anxiety. Mean ± s.e.m., Unpaired two-tailed Student’s *t*-test; ***p < 0.001. See also Supplementary Figs. 10–11.

We next examined the impact of *Rnd2* suppression in adult-born DGNs on anxiety-like behavior by measuring avoidance responses to potentially threatening situations, such as open and bright environments. Using the open field test, we found, four weeks after retrovirus injection, that *Rnd2*-deficient mice showed comparable locomotor activity compared to control mice (Supplementary Fig. 11A). In this test, the time spent in the center was not significantly different between the two groups (Supplementary Fig. 11B, *t*_25_ = 0.64, *p* = 0.53). Following this test, mice were subjected to the emergence test using a dark cylinder placed into a bright open field (Supplementary Fig. 11C–E). We observed no significant difference between groups although the latency to emerge from the reassuring cylinder tends to be longer in *Rnd2*-deficient mice compared to control mice (Supplementary Fig. 11D, *t*_25_ = 1.73, *p* = 0.10). In contrast, in the elevated plus maze (EPM), *Rnd2*-deficient mice (GFP/Cre) spent significantly less time in the open arms, which are threatening areas, compared to control mice (GFP) (EPM1, Fig. 6A, *t*_24_ = 2.48, *p* = 0.02). Interestingly, the reduction of time spent by GFP/Cre-infused mice in the open arms of the EPM remained significantly decreased compared with control mice as long as 10 weeks after virus injection, suggesting a long-lasting effect of the deletion (EPM2, Fig. 6A; *t*_25_ = 2.19, *p* = 0.04). Importantly, this effect was not due to a modification of locomotor activity and/or exploration since the number of total entries was similar between the two groups (Fig. 6B). It is worth noting that, in EPM2, the anxiety level of both groups was increased compared to EPM1, which can be caused by previous exposure to the MWM, or, alternatively, by repeated exposure to the EPM testing apparatus. *Rnd2*-deficient mice also exhibited a significant increase in anxiety-like behavior when subject to a light/dark test. In this test, *Rnd2*-deleted mice escaped more quickly from the illuminated chamber (Fig. 6C, *t*_18_ = 2.80, *p* = 0.01) and spent significantly less time in this chamber compared to control mice (Fig. 6D, *t*_18_ = 4.63, *p* < 0.001).

In a subsequent analysis, a z-normalization was applied [41]. This methodology standardizes observations obtained at different times and from different cohorts, thus allowing their compilation. For each anxiety-like behavior, a Z-score was calculated (see Methods). The directionality of scores was adjusted such that increased score values reflect increased anxiety. Then the z values obtained for each test were averaged to obtain a single z-anxiety score. As shown in Fig. 6E*(*t_45_ = 4.01, *p* < 0.001), this score is significantly increased in mice injected with the GFP/Cre retrovirus compared to control mice further supporting that the suppression of *Rnd2* in adult-born DGNs increases anxiety-like behavior.

In addition to studying anxiety behavior, we also analyzed depression-like behavior. Using a sucrose preference test, we found that the total liquid intake, as well as the consumption of sucrose, were similar between the two groups indicating that the deletion of *Rnd2* does not induce anhedonia-like behavior (Supplementary Fig. 11F, G). Next, we assessed immobility during exposure to inescapable stress using the forced-swim test (FST). In this test, we found that both the latency to immobility and the total time spent floating were not significantly modified by the suppression of *Rnd2*, despite a trend for immobility time (Supplementary Fig. 11H-I, *t*_18_ = 1.92, *p* = 0.07). When Z-score normalization was performed (Supplementary Fig. 11J), we found no significant difference between the two groups suggesting that the absence of *Rnd2* in newborn neurons of the adult DG does not impact depression-like behavior.

Finally, the position and the number of adult-born DGNs targeted by our retroviral approach were quantified at the end of the behavioral sequences (Supplementary Fig. 11K–N). In both batches of animals, transduced cells were located along the septo-temporal axis of the DG and we confirmed that the deletion of *Rnd2* increases the death of newborn neurons in the adult DG. Indeed, fewer GFP+ cells were detected in mice injected with the GFP/Cre retrovirus compared to control mice injected with the GFP retrovirus, although similar titers of virus were infused (see Methods). However, this effect on cell death has no impact on the total volume of the GCL (Supplementary Fig. 11O). Altogether, these experiments demonstrate that *Rnd2* deletion in adult-born DGNs via a retroviral approach impacts anxiety-like behavior but does not affect memory and depression-like behavior.

### Rnd2 plays distinct functions in developmentally and adult-born DGNs

**Fig. 7:**
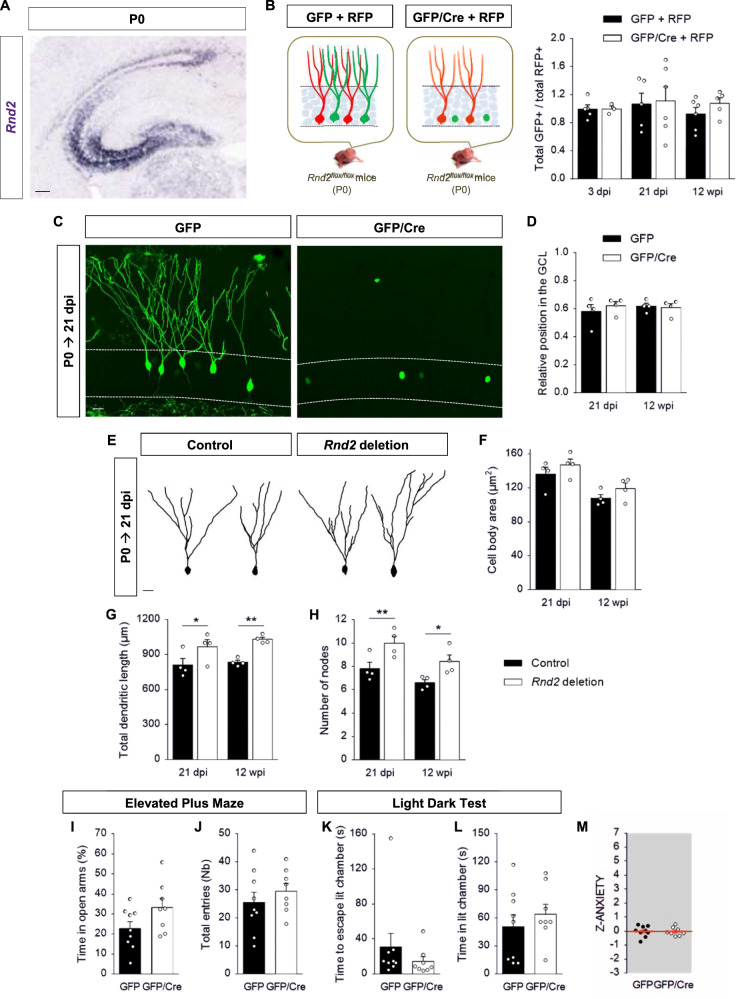
Rnd2 suppression in neonatally-born DGNs only affects their dendritic development. **A** Distribution of *Rnd2* transcripts in the mouse hippocampus at P0. **B** Analysis of the ratio of total GFP+ to total RFP+ cells in *Rnd2*^*flox/flox*^ mice after injection at P0 of a mix of GFP and RFP expressing retroviruses or GFP/Cre and RFP expressing retroviruses. Mean ± s.e.m, *n* = 3–6 mice. **C** Representative images and (**D**) quantification of the relative position of GFP+ neurons within the GCL, 21 days after GFP or GFP/Cre retrovirus injection at P0. Mean ± s.e.m. (*n* = 4 mice). **E** Representative tracings of control and *Rnd2*-deleted neonatally-born DGNs at 21 dpi. **F**–**H** Quantification of the cell body area (**F**), the total dendritic length (**G**) and the number of nodes (**H**) 21 days after P0 injection. Mean ± s.e.m.; paired two-tailed Student’s *t*-test; *p < 0.05, **p < 0.01 (*n* = 4 mice, a minimum of three cells were analyzed per animal). **I** Time spent in the open arms (in % of time spent in the open arms and in the closed arms) and (**J**) number of total entries in the elevated plus maze. **K** Time to escape and (**L**) time spent in the lit area in the light/dark test. **M** z-anxiety score calculated by averaging z-score values of the different tests used to assess anxiety-like behavior. Mean ± s.e.m., GFP *n* = 9 and GFP/Cre *n* = 8 mice. Scale bars represent 100 µm (**A**) and 20 µm (**C**, **E**). See also Supplementary Figs. 12–13.

Lastly, we asked whether the functions of Rnd2 described so far are specific to adult-born DGNs or, in other words, whether Rnd2 plays similar roles in developmentally-born DGNs. To address this, *Rnd2* was deleted using a retroviral approach identical to the one used in adult mice but in this case, GFP or GFP/Cre retroviruses were injected at P0 when generation of DGNs reaches a peak in mice [57, 58] (Supplementary Fig. 12A–C). Importantly, *Rnd2* is prominently expressed at this stage in the DG (Fig. 7A). Since most defects were observed at 21 dpi in adult-born DGNs, a similar time point of analysis was used in this set of experiments. Moreover, we only focused on the cellular processes that were affected in adult-born DGNs, i.e. survival, positioning, and somatodendritic morphogenesis. First, to study the impact of the deletion on cell survival, we looked at caspase-3a expression. Although we were able to detect some caspase-3a+ cells in the DG at 21 dpi (Supplementary Fig. 12D), we never detected any GFP cells expressing this apoptotic marker in both conditions (at least 100 GFP+ cells from 3-4 animals were analyzed in each group). We also performed this analysis at a later time point, 12 weeks post injection (wpi), since it has been suggested that developmentally-born DGNs, in contrast to adult-born DGNs, do not go through a period of cell death during their immature stages but instead die after reaching maturity, between two and six months of age [59]. However, even at this stage, no control or *Rnd2*-deleted GFP+ cells were found to express caspase-3a (data not shown). We then completed these data with a retroviral co-injection strategy. Since the number of transduced cells is rather variable after P0 retrovirus injection, we implemented a different strategy than the one used for adult-born DGNs (Fig. 2E). We co-injected P0 *Rnd2*^*flox/flox*^ pups either with a mixture of two control retroviruses expressing GFP and RFP or with GFP/Cre and RFP expressing retroviruses. The ratio of total GFP+ to total RFP+ cells, which is independent of the total number of labeled cells, was then quantified for each animal in each group at different time points and normalized to the ratios at 3 dpi. Importantly, for each type of virus, the same preparation was used and the same mixture of the two viruses was done for all time points analyzed. Moreover, we validated this strategy with adult-born DGNs (Supplementary Fig. 12E). When injected at P0, the ratio of total GFP+ to total RFP+ labeled cells in the control group (black bars, Fig. 7B) remained constant over time, indicating a similar survival of newborn cells transduced with either control virus. Similarly in the *Rnd2*-knockout group (white bars, Fig. 7B), the ratio remained unchanged across the different time points post-injection indicating that Cre expression does not perturb the viability of the cells. Based upon these observations, we concluded that the survival of P0-born DGNs is not significantly affected by the absence of *Rnd2*, a finding which contrasts the effect of *Rnd2* deletion in adult-born DGNs.

We looked further in these studies and found that the final position of this population of neurons was not impacted by *Rnd2* suppression (Fig. 7C, D). For morphological analysis, RFP and GFP/Cre retroviruses were co-injected at P0 similarly to our experiments in adult mice. Through this analysis, we found that the size of the cell body was not significantly different between the two groups at 21 dpi and 12 wpi (Fig. 7E, F). However, the total dendritic length (Fig. 7E, G; *t*_3_ = 4.06, *p* = 0.03 at 21 dpi; *t*_3_ = 6.26, *p* = 0.008 at 12 wpi), as well as the number of nodes (Fig. 7E, H; *t*_3_ = 7.33, *p* = 0.005 at 21 dpi; *t*_3_ = 3.57, *p* = 0.04 at 12 wpi), were both significantly increased in *Rnd2*-depleted neurons compared to control neurons, when measured at both 21 dpi and 12 wpi. Finally, we assessed whether *Rnd2* deletion in neonatally-born DGNs also affects anxiety-like behavior. For this goal, P0 *Rnd2*^*flox/flox*^ pups were injected bilaterally with Cre/GFP or GFP retrovirus and tested, once they reached adulthood, using the same behavioral tasks as those described for our aforementioned studies of adult-born DGNs (Supplementary Fig. 13A). Interestingly, the suppression of *Rnd2* in neonatally-born DGNs has no impact on anxiety-related behavior (Fig. 7I–M, Supplementary Fig. 13B–F), suggesting that Rnd2 has specific and unique functions in DGNs generated during adulthood. To conclude, in neonatally-born DGNs, Rnd2 seems to be crucial only for dendrite morphogenesis, indicating that granule neurons in the DG exhibit a differential dependency to Rnd2 according to the age and the environment of the hippocampal neurogenic niche.

## Discussion

Despite the central role of Rho GTPases in neuronal development [15], their function in adult neurogenesis has been poorly investigated [16] and the role in particular of the Rnd subfamily is totally unknown. In this study, by using an approach of loss of function, we provide evidence that Rnd2 is essential for AHN and highlight new important functions for this atypical Rho GTPase in vivo. At the cellular level, we demonstrate that Rnd2 is cell-intrinsically required for the survival and maturation of adult-born DGNs. Importantly, these functions seem to be specific to Rnd2 and not shared by the two other Rnd. Indeed, although we did not evaluate their proper contribution to AHN, *Rnd1* and *Rnd3* have distinct expression patterns in the adult DG (Supplementary Fig. 1) and RNAseq data indicate that *Rnd1* and *Rnd3* are not expressed in the same cell types as *Rnd2* in the DG of young mice (http://linnarssonlab.org/dentate/) [43]. Whereas *Rnd2* is particularly enriched in neuroblasts, *Rnd1* is mainly expressed in immature granule neurons and *Rnd3* in intermediate progenitor cells. Moreover, Rnd1 and Rnd3 have been shown, in different cell types including neurons, to be predominantly expressed at the plasma membrane whereas Rnd2 is mainly located in endosomes [23, 60]. Altogether this data supports our hypothesis that Rnd2 may have unique functions in adult-born DGNs.

Our set of experiments with the inducible Cre (Fig. 4, Supplementary Fig. 8) suggests that the functions of Rnd2 in newborn neuron survival and maturation are mediated by distinct mechanisms. The signaling pathways mediating Rnd2 action are still poorly understood [19]. In cortical neurons, during embryogenesis, Rnd2 promotes migration partially through the inhibition of RhoA [23], and pharmacological inhibition of RhoA signaling was shown to enhance the survival of adult-born DGNs [18]. However, we found that the excess death of *Rnd2*-deleted adult-born DGNs was not counteracted by co-delivery of DN-RhoA, suggesting that the action of Rnd2 on adult newborn survival does not involve RhoA inhibition (Supplementary Fig. 6B,C). However, we cannot exclude that RhoA might be part of the mechanisms through which Rnd2 regulates morphological maturation and positioning. Other candidate interactors such as the p38 mitogen-activated protein kinase (MAPK) or Plexins might be relevant to the functions of Rnd2 in adult-born DGNs. Indeed, Rnd2 attenuates apoptosis and autophagy in glioblastoma cells by targeting the p38 MAPK signaling pathway [61]. Regarding Plexins, the semaphorin receptors, have been implicated in AHN [62, 63], especially in migration and dendritic growth, and have been shown to be bound and regulated by Rnd2 in neurons and other cell types [37, 64, 65]. Whether Plexins or p38 mediate some aspects of Rnd2 action in adult newborn neurons remains to be investigated. In addition, as already mentioned, Rnd2 has been shown to be expressed in endosomes [23, 60, 66] and to interact with molecules involved in the formation and trafficking of endocytic vesicles [66, 67]. This raises the possibility that Rnd2 activity in adult newborn neurons may involve endocytosis and, in particular, Rnd2 may regulate the trafficking of membrane-associated molecules such as receptors or adhesion molecules that control survival, migration, and/or morphological development. Among these molecules, the NMDA receptor could be a candidate. Indeed, like Rnd2, NMDA receptors are critical for the survival of immature neurons at the time of their integration into the pre-existing neuronal network [12].

Our data also reveal that Rnd2 has distinct functions during adult and developmental neurogenesis in the DG since its deletion in DGNs generated at birth only affects their dendritic arborization. These results argue against the view that adult neurogenesis is a simple continuation of developmental neurogenesis and further highlight differences between the two processes. Indeed, although adult neurogenesis recapitulates the entire process of neuronal development that occurs during embryonic or early postnatal stages, fundamental differences are evident between development and adulthood [68, 69]. For example, in the developing brain, nascent neurons must cope with a continuously changing environment, while their counterparts in adult brain are surrounded by a relatively stable niche. More specifically in the DG, adult-born DGNs and developmentally-born DGNs exhibit distinct morphological features once mature [31, 70], undergo distinct survival dynamics [59], have differential ability to reshape in response to a learning experience [71, 72], are recruited during different learning tasks [73–75] and are required for different functions [53, 76, 77]. How Rnd2 plays distinct functions in developmentally-born and adult-born DGNs remains to be elucidated. The absence of specific interactors/effectors of Rnd2 during development might be part of the answer. However, it cannot be excluded that the difference in survival for example may just reflect the concept that the successful survival of adult-born neurons is input dependent, which may not be the case for developmentally-born neurons. Another possibility could be that Rnd1 and/or Rnd3 compensate for *Rnd2* deletion at P0 but not during adulthood.

In addition to cellular effects, we show that *Rnd2* deletion in adult-born DGNs impacts anxiety-like behavior while depression-like behavior is not affected. These results reproduce what we and others have obtained following the depletion of new DGNs in the adult brain [55, 78]. In contrast, in our study, reference memory and behavioral pattern separation are not altered. These results were unexpected since we showed that these processes are impaired in the Nestin-rtTA/TRE-Bax mice [51, 54]. Several factors may explain these distinct results, including differences in the experimental design of behavior assays or the method used to target adult newborn neurons. For example, the retroviral strategy only targets a population of new neurons born at a specific time whereas the previous approach, using doxycycline for ablation, targets a broader population. So memory processes might be affected only when a large population of adult newborn DGNs is suppressed. In accordance with this assumption, it was shown that an extensive lesion of the hippocampus is required to observe memory deficits [79]. Consequently, anxiety-like behavior may be more sensitive to the loss of new neurons compared to memory processes, hence the behavior of *Rnd2-*deficient mice in the present study. An alternative explanation of our phenotype is that Rnd2 might play a more prominent role in adult-born DGNs located in the temporal DG. This hypothesis is based on several lines of evidence. Firstly, memory function depends on the septal hippocampus whereas anxiety is related to its temporal part [80, 81]. Secondly, adult-born DGNs in the temporal DG seem to be especially important in stress-induced regulation of anxiety [82]. Thirdly, *Rnd2* expression is particularly enriched in the temporal DG (Fig. 1E and http://hipposeq.janelia.org).

Overall, although it is not possible to determine whether the effects on anxiety are directly due to the loss of *Rnd2* in adult newborn neurons or just to the decreased numbers of these cells, our findings demonstrate that Rnd2 is cell-autonomously required for the proper development of adult-born DGNs and serves a critical role in the regulation of anxiety-like behavior from the neurogenic niche. These data validate not only the neurogenesis hypothesis of anxiety but also identify Rnd2 as a molecular link between AHN and anxiety.

## Supplementary information

Supplementary information

## References

[1] Goncalves JT, Schafer ST, Gage FH (2016). Adult neurogenesis in the Hippocampus: from stem. Cells Behav Cell.

[2] Altman J, Bayer SA (1990). Migration and distribution of two populations of hippocampal granule cell precursors during the perinatal and postnatal periods. J Comp Neurol.

[3] Schlessinger AR, Cowan WM, Gottlieb DI (1975). An autoradiographic study of the time of origin and the pattern of granule cell migration in the dentate gyrus of the rat. J Comp Neurol.

[4] Boldrini M, Fulmore CA, Tartt AN, Simeon LR, Pavlova I, Poposka V (2018). Human Hippocampal neurogenesis persists throughout aging. cell stem cell.

[5] Eriksson PS, Perfilieva E, Bjork-Eriksson T, Alborn AM, Nordborg C, Peterson DA (1998). Neurogenesis in the adult human hippocampus. Nat Med.

[6] Moreno-Jiménez EP, Flor-García M, Terreros-Roncal J, Rábano A, Cafini F, Pallas-Bazarra N, et al. Adult hippocampal neurogenesis is abundant in neurologically healthy subjects and drops sharply in patients with Alzheimer’s disease. Nature medicine 2019. 10.1038/s41591-019-0375-9 30911133

[7] Spalding KL, Bergmann O, Alkass K, Bernard S, Salehpour M, Huttner HB (2013). Dynamics of hippocampal neurogenesis in adult humans. Cell.

[8] Anacker C, Hen R (2017). Adult hippocampal neurogenesis and cognitive flexibility - linking memory and mood. Nat Rev Neurosci.

[9] Abrous DN, Koehl M, Le Moal M (2005). Adult neurogenesis: from precursors to network and physiology. Physiol Rev.

[10] Sun GJ, Sailor KA, Mahmood QA, Chavali N, Christian KM, Song H (2013). Seamless reconstruction of intact adult-born neurons by serial end-block imaging reveals complex axonal guidance and development in the adult hippocampus. J Neurosci: Off J Soc Neurosci.

[11] Kempermann G, Gast D, Kronenberg G, Yamaguchi M, Gage FH (2003). Early determination and long-term persistence of adult-generated new neurons in the hippocampus of mice. Development.

[12] Tashiro A, Sandler VM, Toni N, Zhao C, Gage FH (2006). NMDA-receptor-mediated, cell-specific integration of new neurons in adult dentate gyrus. Nature.

[13] Toda T, Parylak SL, Linker SB, Gage FH. The role of adult hippocampal neurogenesis in brain health and disease. Mol psychiatry. 2018;24:67–87. 10.1038/s41380-018-0036-2 PMC6195869 29679070

[14] Govek EE, Hatten ME, Van, Aelst L (2011). The role of Rho GTPase proteins in CNS neuronal migration. Developmental Neurobiol.

[15] Govek EE, Newey SE, Van, Aelst L (2005). The role of the Rho GTPases in neuronal development. Genes Dev.

[16] Vadodaria KC, Jessberger S (2013). Maturation and integration of adult born hippocampal neurons: signal convergence onto small Rho GTPases. Front synaptic Neurosci.

[17] Vadodaria KC, Brakebusch C, Suter U, Jessberger S (2013). Stage-specific functions of the small Rho GTPases Cdc42 and Rac1 for adult hippocampal neurogenesis. J Neurosci: Off J Soc Neurosci.

[18] Christie KJ, Turbic A, Turnley AM (2013). Adult hippocampal neurogenesis, Rho kinase inhibition and enhancement of neuronal survival. Neuroscience.

[19] Basbous S, Azzarelli R, Pacary E, Moreau V. Pathophysiological functions of Rnd proteins. Small GTPases 2020;1–22 10.1080/21541248.2020.1829914. Online ahead of print. 10.1080/21541248.2020.1829914 PMC8583191 33054516

[20] Grise F, Bidaud A, Moreau V (2009). Rho GTPases in hepatocellular carcinoma. Biochimica et biophysica acta.

[21] Miller JA, Nathanson J, Franjic D, Shim S, Dalley RA, Shapouri S (2013). Conserved molecular signatures of neurogenesis in the hippocampal subgranular zone of rodents and primates. Development.

[22] Heng JI, Nguyen L, Castro DS, Zimmer C, Wildner H, Armant O (2008). Neurogenin 2 controls cortical neuron migration through regulation of Rnd2. Nature.

[23] Pacary E, Heng J, Azzarelli R, Riou P, Castro D, Lebel-Potter M (2011). Proneural transcription factors regulate different steps of cortical neuron migration through Rnd-mediated inhibition of RhoA signaling. Neuron.

[24] Azzarelli R, Guillemot F, Pacary E (2015). Function and regulation of Rnd proteins in cortical projection neuron migration. Front Neurosci.

[25] Fujita H, Katoh H, Ishikawa Y, Mori K, Negishi M (2002). Rapostlin is a novel effector of Rnd2 GTPase inducing neurite branching. J Biol Chem.

[26] Tanaka H, Katoh H (2006). Negishi M. Pragmin, a novel effector of Rnd2 GTPase, stimulates RhoA activity. J Biol Chem.

[27] Zhao C, Teng EM, Summers RG, Ming GL, Gage FH (2006). Distinct morphological stages of dentate granule neuron maturation in the adult mouse hippocampus. J Neurosci: Off J Soc Neurosci.

[28] Laplagne DA, Esposito MS, Piatti VC, Morgenstern NA, Zhao C, van Praag H (2006). Functional convergence of neurons generated in the developing and adult hippocampus. PLoS Biol.

[29] Mu Y, Zhao C, Toni N, Yao J, Gage FH (2015). Distinct roles of NMDA receptors at different stages of granule cell development in the adult brain. eLife.

[30] Heinrich C, Gascon S, Masserdotti G, Lepier A, Sanchez R, Simon-Ebert T (2011). Generation of subtype-specific neurons from postnatal astroglia of the mouse cerebral cortex. Nat Protoc.

[31] Kerloch T, Clavreul S, Goron A, Abrous DN, Pacary E. Dentate granule neurons generated during perinatal life display distinct morphological features compared with later-born neurons in the mouse Hippocampus. Cereb cortex. 2018;29:3527–39. 10.1093/cercor/bhy224 30215686

[32] Boutin C, Diestel S, Desoeuvre A, Tiveron MC, Cremer H (2008). Efficient in vivo electroporation of the postnatal rodent forebrain. PLoS One.

[33] Herzog E, Gilchrist J, Gras C, Muzerelle A, Ravassard P, Giros B (2004). Localization of VGLUT3, the vesicular glutamate transporter type 3, in the rat brain. Neuroscience.

[34] Bianchi P, Ciani E, Guidi S, Trazzi S, Felice D, Grossi G (2010). Early pharmacotherapy restores neurogenesis and cognitive performance in the Ts65Dn mouse model for Down syndrome. J Neurosci: Off J Soc Neurosci.

[35] Rodriguez A, Ehlenberger DB, Dickstein DL, Hof PR, Wearne SL (2008). Automated three-dimensional detection and shape classification of dendritic spines from fluorescence microscopy images. PLoS One.

[36] Bolos M, Terreros-Roncal J, Perea JR, Pallas-Bazarra N, Avila J, Llorens-Martin M (2019). Maturation dynamics of the axon initial segment (AIS) of newborn dentate granule cells in young adult C57BL/6 J mice. J Neurosci: Off J Soc Neurosci.

[37] Azzarelli R, Pacary E, Garg R, Garcez P, van den Berg D, Riou P (2014). An antagonistic interaction between PlexinB2 and Rnd3 controls RhoA activity and cortical neuron migration. Nat Commun.

[38] Babu H, Claasen JH, Kannan S, Runker AE, Palmer T, Kempermann G (2011). A protocol for isolation and enriched monolayer cultivation of neural precursor cells from mouse dentate gyrus. Front Neurosci.

[39] Bustin SA, Benes V, Garson JA, Hellemans J, Huggett J, Kubista M (2009). The MIQE guidelines: minimum information for publication of quantitative real-time PCR experiments. Clin Chem.

[40] Livak KJ, Schmittgen TD (2001). Analysis of relative gene expression data using real-time quantitative PCR and the 2(-Delta Delta C(T)) Method. Methods.

[41] Guilloux JP, Seney M, Edgar N, Sibille E (2011). Integrated behavioral z-scoring increases the sensitivity and reliability of behavioral phenotyping in mice: relevance to emotionality and sex. J Neurosci methods.

[42] Cembrowski MS, Wang L, Sugino K, Shields BC, Spruston N (2016). Hipposeq: a comprehensive RNA-seq database of gene expression in hippocampal principal neurons. eLife.

[43] Hochgerner H, Zeisel A, Lonnerberg P, Linnarsson S (2018). Conserved properties of dentate gyrus neurogenesis across postnatal development revealed by single-cell RNA sequencing. Nat Neurosci.

[44] Tashiro A, Zhao C, Gage FH (2006). Retrovirus-mediated single-cell gene knockout technique in adult newborn neurons in vivo. Nat Protoc.

[45] Savill J (1997). Recognition and phagocytosis of cells undergoing apoptosis. Br Med Bull.

[46] Sierra A, Encinas JM, Deudero JJ, Chancey JH, Enikolopov G, Overstreet-Wadiche LS (2010). Microglia shape adult hippocampal neurogenesis through apoptosis-coupled phagocytosis. cell stem cell.

[47] Esposito MS, Piatti VC, Laplagne DA, Morgenstern NA, Ferrari CC, Pitossi FJ (2005). Neuronal differentiation in the adult hippocampus recapitulates embryonic development. J Neurosci: Off J Soc Neurosci.

[48] Duan X, Chang JH, Ge S, Faulkner RL, Kim JY, Kitabatake Y (2007). Disrupted-In-Schizophrenia 1 regulates integration of newly generated neurons in the adult brain. Cell.

[49] Mainen ZF, Sejnowski TJ (1996). Influence of dendritic structure on firing pattern in model neocortical neurons. Nature.

[50] van Elburg RA, van Ooyen A (2010). Impact of dendritic size and dendritic topology on burst firing in pyramidal cells. PLoS computational Biol.

[51] Dupret D, Revest JM, Koehl M, Ichas F, De Giorgi F, Costet P (2008). Spatial relational memory requires hippocampal adult neurogenesis. PLoS One.

[52] Clelland CD, Choi M, Romberg C, Clemenson GD, Fragniere A, Tyers P (2009). A functional role for adult hippocampal neurogenesis in spatial pattern separation. Science.

[53] Nakashiba T, Cushman JD, Pelkey KA, Renaudineau S, Buhl DL, McHugh TJ (2012). Young dentate granule cells mediate pattern separation, whereas old granule cells facilitate pattern completion. Cell.

[54] Tronel S, Belnoue L, Grosjean N, Revest JM, Piazza PV, Koehl M, et al. Adult-born neurons are necessary for extended contextual discrimination. Hippocampus. 2010;22:292–8. 10.1002/hipo.20895 21049483

[55] Revest JM, Dupret D, Koehl M, Funk-Reiter C, Grosjean N, Piazza PV (2009). Adult hippocampal neurogenesis is involved in anxiety-related behaviors. Mol psychiatry.

[56] Koehl M, Abrous DN (2011). A new chapter in the field of memory: adult hippocampal neurogenesis. Eur J Neurosci.

[57] Angevine JB, Jr. Time of neuron origin in the hippocampal region. An autoradiographic study in the mouse. Exp neurol Suppl. 1965: Suppl 2:1–70. 5838955

[58] Snyder JS (2019). Recalibrating the relevance of adult neurogenesis. Trends Neurosci.

[59] Cahill SP, Yu RQ, Green D, Todorova EV, Snyder JS (2017). Early survival and delayed death of developmentally-born dentate gyrus neurons. Hippocampus.

[60] Roberts PJ, Mitin N, Keller PJ, Chenette EJ, Madigan JP, Currin RO (2008). Rho Family GTPase modification and dependence on CAAX motif-signaled posttranslational modification. J Biol Chem.

[61] Xu Y, Sun Q, Yuan F, Dong H, Zhang H, Geng R (2020). RND2 attenuates apoptosis and autophagy in glioblastoma cells by targeting the p38 MAPK signalling pathway. J Exp Clin cancer Res: CR.

[62] Jongbloets BC, Lemstra S, Schellino R, Broekhoven MH, Parkash J, Hellemons AJ (2017). Stage-specific functions of Semaphorin7A during adult hippocampal neurogenesis rely on distinct receptors. Nat Commun.

[63] Zhao XF, Kohen R, Parent R, Duan Y, Fisher GL, Korn MJ (2018). PlexinA2 forward signaling through Rap1 GTPases regulates dentate gyrus development and schizophrenia-like behaviors. Cell Rep.

[64] McColl B, Garg R, Riou P, Riento K, Ridley AJ (2016). Rnd3-induced cell rounding requires interaction with Plexin-B2. J cell Sci.

[65] Uesugi K, Oinuma I, Katoh H, Negishi M (2009). Different requirement for Rnd GTPases of R-Ras GAP activity of Plexin-C1 and Plexin-D1. J Biol Chem.

[66] Tanaka H, Fujita H, Katoh H, Mori K, Negishi M (2002). Vps4-A (vacuolar protein sorting 4-A) is a binding partner for a novel Rho family GTPase, Rnd2. Biochemical J.

[67] Kamioka Y, Fukuhara S, Sawa H, Nagashima K, Masuda M, Matsuda M (2004). A novel dynamin-associating molecule, formin-binding protein 17, induces tubular membrane invaginations and participates in endocytosis. J Biol Chem.

[68] Gotz M, Nakafuku M, Petrik D. Neurogenesis in the developing and adult brain-similarities and key differences. Cold Spring Harbor perspectives in biol. 2016;8. 10.1101/cshperspect.a018853 PMC4930921 27235475

[69] Urban N, Guillemot F (2014). Neurogenesis in the embryonic and adult brain: same regulators, different roles. Front Cell Neurosci.

[70] Cole JD, Espinueva D, Seib DR, Ash AM, Cooke MB, Cahill SP, et al. Adult-born hippocampal neurons undergo extended development and are morphologically distinct from neonatally-born neurons Prolonged development of adult-born neurons. The J of neurosci. 2020;40:5740–56. 10.1523/JNEUROSCI.1665-19.2020 PMC7380968 32571837

[71] Lemaire V, Tronel S, Montaron MF, Fabre A, Dugast E, Abrous DN (2012). Long-lasting plasticity of hippocampal adult-born neurons. J Neurosci: Off J Soc Neurosci.

[72] Tronel S, Fabre A, Charrier V, Oliet SH, Gage FH, Abrous DN (2010). Spatial learning sculpts the dendritic arbor of adult-born hippocampal neurons. Proc Natl Acad Sci USA.

[73] Lods M, Pacary E, Mazier W, Farrugia F, Mortessagne P, Masachs N, et al. Adult-born neurons immature during learning are necessary for remote memory reconsolidation in rats. Nat commun. 2021;12:1778. 10.1038/s41467-021-22069-4 PMC7979763 33741954

[74] Montaron MF, Charrier V, Blin N, Garcia P, Abrous DN (2020). Responsiveness of dentate neurons generated throughout adult life is associated with resilience to cognitive aging. Aging cell.

[75] Tronel S, Lemaire V, Charrier V, Montaron MF, Abrous DN (2015). Influence of ontogenetic age on the role of dentate granule neurons. Brain Struct Funct.

[76] Abrous DN, Koehl M, Lemoine MA. Baldwin interpretation of adult hippocampal neurogenesis: from functional relevance to physiopathology. Mol psychiatry. 2021. 10.1038/s41380-021-01172-4. Online ahead of print. 10.1038/s41380-021-01172-4 PMC8960398 34103674

[77] Wei L, Meaney MJ, Duman RS, Kaffman A (2011). Affiliative behavior requires juvenile, but not adult neurogenesis. J Neurosci: Off J Soc Neurosci.

[78] Bergami M, Rimondini R, Santi S, Blum R, Gotz M, Canossa M (2008). Deletion of TrkB in adult progenitors alters newborn neuron integration into hippocampal circuits and increases anxiety-like behavior. Proc Natl Acad Sci USA.

[79] Moser MB, Moser EI, Forrest E, Andersen P, Morris RG (1995). Spatial learning with a minislab in the dorsal hippocampus. Proc Natl Acad Sci USA.

[80] Bannerman DM, Rawlins JN, McHugh SB, Deacon RM, Yee BK, Bast T (2004). Regional dissociations within the hippocampus-memory and anxiety. Neurosci Biobehav Rev.

[81] Fanselow MS, Dong HW (2010). Are the dorsal and ventral hippocampus functionally distinct structures?. Neuron.

[82] Anacker C, Luna VM, Stevens GS, Millette A, Shores R, Jimenez JC (2018). Hippocampal neurogenesis confers stress resilience by inhibiting the ventral dentate gyrus. Nature.

