# Supplementary material for: The atypical Rho GTPase Rnd2 is critical for dentate granule neuron development and anxiety-like behavior during adult but not neonatal neurogenesis
Source: Mol Psychiatry. 2021 Sep 24;26(12):7280–95. doi: 10.1038/s41380-021-01301-z (PMC8872985; doi:10.1038/s41380-021-01301-z)
Supplement: Supplementary file 1 — Supplementary information [file 41380_2021_1301_MOESM1_ESM.pdf]

SUPPLEMENTARY INFORMATION

The atypical Rho GTPase Rnd2 is critical for dentate granule neuron development and anxiety-like behavior during adult but not neonatal neurogenesis

Thomas Kerloch [1], Fanny Farrugia [1], Lou Bouit [2], Marlène Maître [3], Geoffrey Terral [1], Muriel Koehl [1], Pierre Mortessagne [1], Julian Ik-Tsen Heng [4], Mylène Blanchard [1], Hélène Doat [3,5], Thierry Leste-Lasserre [5], Adeline Goron [1], Delphine Gonzales [6], David Perrais [2], François Guillemot [7], Djoher Nora Abrous [1,8], Emilie Pacary [1,8*]

SUPPLEMENTARY FIGURE LEGENDS

Supplementary Figure 1: *Rnd1* and *Rnd3* expressions in the mouse DG.

(**A**) Sequential images of the hippocampus showing the region (SGZ and GCL) microdissected by laser capture from cresyl violet-stained sections. (**B**) Analysis by real-time PCR of *Rnd1* and *Rnd3* mRNA expressions in microdissected DG (SGZ and GCL) at different ages. Data are presented as fold change compared with the expression level at P7 ± s.e.m (n = 4 mice per time point). P, postnatal day; W, weeks. (**C**) Distribution of *Rnd1* and *Rnd3* transcripts in the adult hippocampus (12-week old mouse). **(D**) Analysis by real-time PCR of *Rnd1* and *Rnd3* mRNA expressions along the septo-temporal axis in the adult (12-week old) microdissected DG. Data are presented as fold change compared with the expression level at the anteroposterior coordinate -1.34 from the Bregma ± s.e.m (n = 5 mice per time point).

Scale bar represents 300 µm (A, C).

Supplementary Figure 2: *Rnd2* in the mouse DG.

(**A**) t-SNE visualization of Dataset A from Hochgerner et al., 2018 (http://linnarssonlab.org/dentate/) stained for *Rnd2*. Grey indicates low expression, red high expression. VLMC, vascular and leptomeningeal cell; PVM, perivascular macrophage; OL, oligodendrocyte; NFOL, newly formed oligodendrocyte; OPC, oligodendrocyte precursor cell; nIPCs, neuronal intermediate progenitor cells.

Supplementary Figure 3: *Rnd2* deletion in adult-born DGNs using *$Rnd2^{flox/flox}$* mice and Cre expressing retrovirus.

(**A**) *Rnd2* conditional mutant allele. This allele contains one loxP site between the exon 1 and 2 and a second loxP site after the last exon. A neomycin (Neo) selection gene flanked by flippase recognition target (FRT) sites was inserted in 3' of *Rnd2*. Following transmission of the mutation to the germline, the Neo gene was excised, giving rise to the *$Rnd2^{flox}$* allele. Upon Cre recombination, exons 2 to 5 of *Rnd2* are deleted. (**B**) Analysis 7 days post-retroviral injection of *Rnd1*, *Rnd2* and *Rnd3* transcripts by quantitative RT-PCR in micro-dissected GFP+ cells. Graphs show expression levels normalized to housekeeping genes and relative to *Rnd* expression in control condition (GFP). (n=9 mice for GFP condition with a total of 6083 GFP+ cells, n=8 for GFP/Cre condition with a total of 5832 GFP+ cells; 561-878 cells per animal). Mean ± s.e.m., Unpaired two-tailed Student's t-test; ***p < 0.001.

Supplementary Figure 4: *Rnd2* suppression in adult hippocampal neural precursor cells. (**A-D**) Adult hippocampal neural precursor cells were prepared from the DG of adult *Rnd2*$^{flox/flox}$ mice. Under proliferative conditions, these cells express the neural precursor cell marker Nestin (B) and after few days of differentiation they generate neurons as attested by the expression of the neuronal markers Tuj1 (C) or MAP2 (D). D0 and D5, differentiation day 0 and 5. (**E**) Analysis by real-time PCR of *Nestin*, *Tuj1* and *Rnd2* mRNA expressions after differentiation of adult hippocampal neural precursor cells. Data are presented as fold change compared with the expression level at D0 ± s.e.m (n = 3 per time point). (**F-G**) Two days after the infection with GFP/Cre or GFP retrovirus, *Rnd2*$^{flox/flox}$ cells were induced to differentiate and the expression of Rnd2 was assessed by immunocytochemistry (**F**) or western blotting (**G**) after 2 days.

Scale bars represent 20 µm (B, C, D) and 10 µm (F).

Supplementary Figure 5: *Rnd2* deletion does not impact the proliferation and neuronal differentiation of adult-born DGNs.

(**A**) Immunostainings for GFP and Ki67 in the DG 3 days after GFP (control) or GFP/Cre (*Rnd2* deletion) retrovirus injection. TOTO labels nuclei (**B**) Quantification of the percentage of transduced cells that are Ki67+ at 3 and 7 days post-injetion (dpi). Mean ± s.e.m (n = 4-6 mice). (**C**) Immunostainings for GFP and doublecortin (DCX) in the DG 14 days after virus injection. (**B**) Quantification of the percentage of transduced cells that are DCX+ at 7, 14 and 21 dpi. Mean ± s.e.m (n = 4-7 mice).

Scale bars represent 20 µm (A, C).

Supplementary Figure 6: The exacerbated cell death and dendritic outgrowth are specific to *Rnd2* suppression.

(**A**) The retroviral co-injection strategy was used in C57Bl6/J mice. The graph shows the survival rate of only RFP+ cells and total GFP+ cells. Mean ± s.e.m.; paired two-tailed Student's t-test; (n = 8 mice for each time point). (**B**) Experimental design for survival rescue experiments. Double stained (DS) cells were quantified 3 and 21 days after injection of a mixture of retroviruses into the DG of adult *Rnd2*$^{flox/flox}$ mice. (**C**) Survival rate of DS cells at 21 days post-injection (dpi). The number of DS cells at 21 dpi was normalized to the number obtained for the corresponding viral mixture at 3 dpi. Mean ± s.e.m.; one way ANOVA followed by Dunnett's multiple comparison test; *$p < 0.05$ compared to Rnd2 rescue (n = 8-

10 mice). (**D-F**) Quantification of the total dendritic length (D), the number of nodes (E) and the cell body area (F) in wild-type (WT) and *Rnd2*$^{flox/flox}$ mice 21 days after the injection of a mixture of GFP/Cre and RFP retroviruses. Mean ± s.e.m.; one way ANOVA followed by Dunnett's multiple comparison test; *p < 0.05 **p<0.01 compared to only RFP+ (*Rnd2*^{flox}) (n = 6-7 mice).

Supplementary Figure 7: *Rnd2* deletion does not have a major impact on dendritic spines, CA3 MFTs and AIS of adult-born DGNs.

(**A**) Representative images of control and *Rnd2*-deleted dendritic segments at 21 and 28 dpi. (**B**, **C**, **D**) Quantification of the total spine density (**B**) and the density of the different spine types at 21 dpi (**C**) and 28 dpi (**D**). Mean ± s.e.m.; paired two-tailed Student's t-test; *p < 0.05 (n = 6 at 21 dpi and n = 4 mice at 28 dpi, a minimum of 3 cells were analyzed per animal). (**E**) Representative image of the AIS (marked with Ankyrin-G immunostaining) of a 28-day-old control (RFP+) adult-born DGN. (**F-H**) AIS length, distance to the soma and thickness in control (RFP+ only) and *Rnd2*-deleted adult-born DGN (GFP+ and RFP+) 28 days after the injection of a mixture of CAG-GFP/Cre and CAG-RFP retroviruses. Mean ± s.e.m, n = 17-28 AIS from 4 animals, a minimum of three AIS was analyzed per animal.

Scale bars represent 5 µm (A, E).

Supplementary Figure 8: *Rnd2* deletion at 28 dpi induces cell death but does affect cell positioning and morphology.

(**A**) A retrovirus expressing GFP together with a conditionally active form of Cre recombinase, which is activated upon tamoxifen, was injected into the DG of adult *Rnd2*$^{flox/flox}$ mice. Four weeks after virus injection, tamoxifen (150 mg/kg, daily for 5 days), or oil in control group, was injected and animals were sacrificed 21 days after the last injection of tamoxifen. (**B**) The relative number of GFP+ cells, the relative position in the GCL and the morphology of transduced cells were quantified 21 days after the last injection of tamoxifen. Mean ± s.e.m., Unpaired two-tailed Student's t-test; *p<0.05 (n=5-8 animals per group).

Supplementary Figure 9: *Rnd2* deletion does not impact the membrane properties of adult-born DGNs.

(**A**) Images of adult newborn neurons transduced with GFP or GFP/Cre retrovirus at 28 dpi in the DG of *Rnd2*$^{flox/flox}$ mice. Biocytin identifies neurons in which whole-cell patch-clamp recording was performed. (**B**, **C**, **D**) Quantification of the resting membrane potential (A),

membrane capacitance (B) and membrane resistance (C). Mean ± s.e.m (n = 30 neurons from 4 mice in GFP group and n = 17 neurons from 8 mice in GFP/Cre group).
Scale bar represents 20 µm (A).

Supplementary Figure 10: *Rnd2* suppression in adult-born DGNs, using a retroviral approach, does not perturb hippocampal-dependent memory.

(**A**) Experimental design and diagram of the behavioral task sequences. (**B**) Experimental design used in the Morris water maze. NW, quadrant north west; NE, quadrant north east; SW, quadrant south west; SE, quadrant south east. (**C**) Latency to reach the hidden platform using variable start positions, 5 weeks after GFP or GFP/Cre retrovirus injection. (**D**) Time spent in the different quadrants during the probe test. (**E**) Experimental design used for contextual fear conditioning and context discrimination. (**F, G**) Contextual fear conditioning assessed by the percentage of freezing when mice were re-exposed (**F**) 24 hours or (**G**) 5 weeks later to the conditioning context (context A) and to a similar context (context B). The percentage of freezing for the baseline was measured during the first three minutes before the first shock. Mean ± s.e.m., two way ANOVA; **p<0.01 ***p<0.001.

Supplementary Figure 11: *Rnd2* suppression in adult-born DGNs increases anxiety-like behavior but does not affect depressive-like behavior.

(**A**) Total travelled distance in the arena and (**B**) time spent in the center of the open field, 4 weeks after GFP or GFP/Cre retrovirus injection. (**C**) Total travelled distance, (**D**) latency to emerge from the cylinder and (**E**) number of re-entries in the cylinder in the emergence test, 4 weeks after virus infusion. (**F**) Sucrose preference (% of total intake) and (**G**) total intake 9 weeks after GFP or GFP/Cre virus injection into $Rnd2^{flox/flox}$ mice. (**H**) Latency to first immobility and (**I**) total immobility time in the forced-swim test, 11 weeks after retrovirus injection. (**J**) Final z-depression score after averaging z-score values of individual test. (**K-N**) Quantification of the number of transduced (GFP+) cells at the end of the behavioral sequences. The number of GFP+ cells along the septo-temporal axis (K, M) is shown as well as the total number per mouse (L, N) for the first (K, L) and second (M, N) batch. (**O**) Total DG volume at the end of the behavioral sequence. All data are presented as the mean ± s.e.m., Unpaired two-tailed Student's t-test; **p<0.01, ***p<0.001. Batch 1 (GFP n=13; GFP/Cre n=14), Batch 2 (GFP n=11; GFP/Cre n=9).

Supplementary Figure 12: In contrast to adult-born DGNs, the survival of P0-born DGNs is not affected by the absence of *Rnd2*.

(A-B) Representative images of adult brains injected at P0 with GFP (A) or GFP/Cre retrovirus (B). (**C**) Retrovirus injection into the lateral ventricle at P0 targets mainly DGNs. Cells with astroglial morphology (white arrowheads) are also observed. (**D**) Immunostaining for GFP and caspase-3a in the DG of $Rnd2^{flox/flox}$ pups, 21 days after GFP or GFP/Cre retrovirus injection at P0. TOTO labels nuclei and identifies the GCL. (**E**) Analysis of the ratio of total GFP+ to total RFP+ cells after injection into the DG of adult $Rnd2^{flox/flox}$ mice of a mix of GFP and RFP expressing retroviruses or GFP/Cre and RFP expressing retroviruses. Mean ± s.e.m, unpaired two-tailed Student's t-test; *$p < 0.05$, n = 7-8 mice.
Scale bars represent 500 µm (A, B), 100 µm (C) and 10 µm (D).

Supplementary Figure 13: *Rnd2* suppression in neonatally-born DGNs does not affect anxiety-like behavior in adult mice.

(**A**) Experimental design and diagram of the behavioral task sequence. The behavioral tasks to measure anxiety-like behavior were performed 16 weeks after GFP or GFP/Cre retrovirus injection into the DG of P0 $Rnd2^{flox/flox}$ pups. (**B**) Total travelled distance in the arena and (**C**) time spent in the center of the open field. (**D**) Total travelled distance, (**E**) latency to emerge from the cylinder and (**F**) number of re-entries in the cylinder in the emergence test. (**G-H**) Quantification of the number of GFP+ cells at the end of the behavioral sequence. The number of GFP+ cells along the septo-temporal axis (H) is shown as well as the total number per mouse (G). (**I**) Total DG volume at the end of the behavioral sequence. Mean ± s.e.m., Unpaired two-tailed Student's t-test, GFP n=9 and GFP/Cre n=8 mice.

FIGURE S1

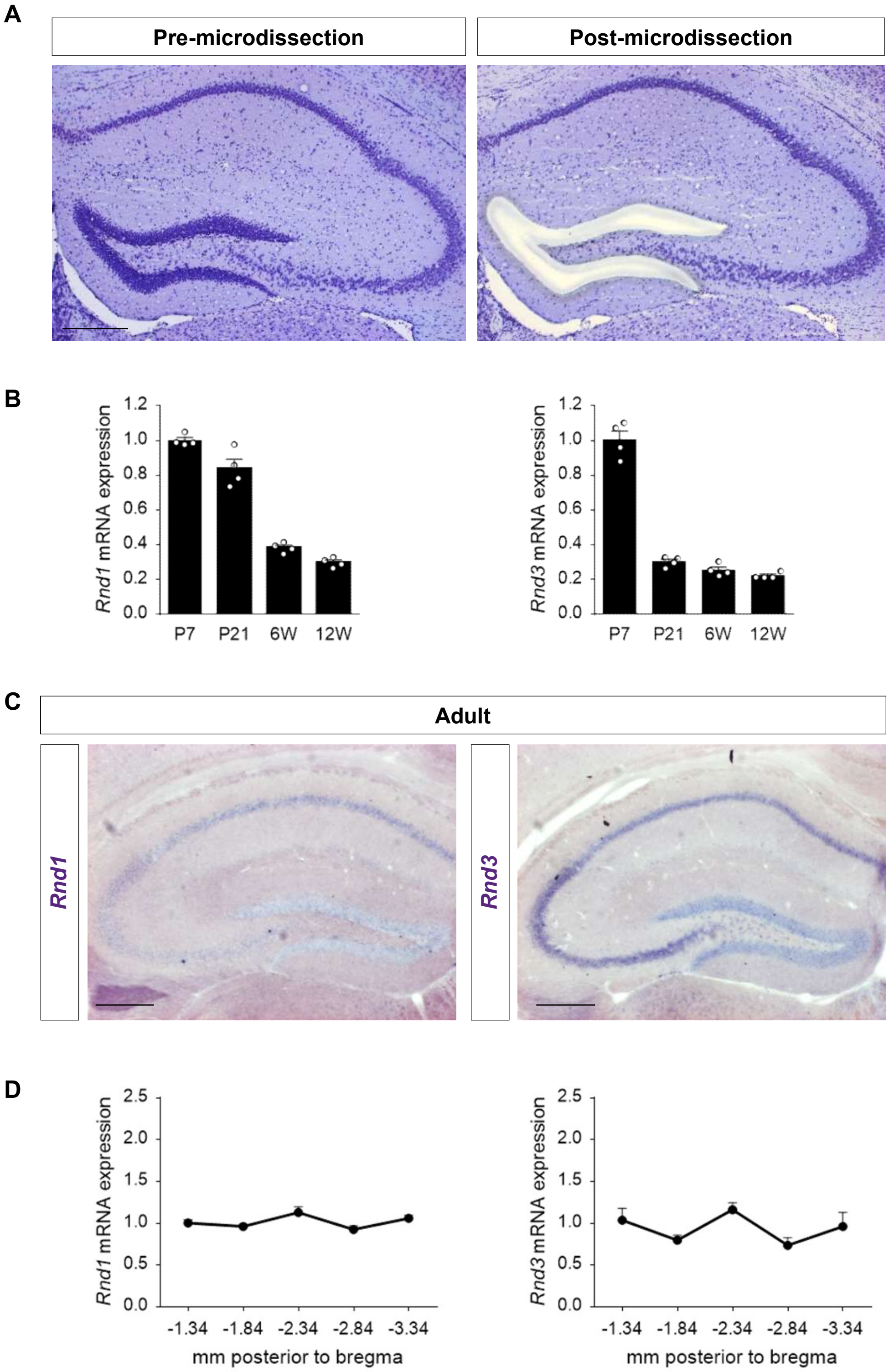
A
Pre-microdissection
Post-microdissection
B
Rnd1 mRNA expression
1.2
1.0
0.8
0.6
0.4
0.2
0.0
P7
P21
6W
12W
Rnd3 mRNA expression
C
Adult
Rnd1
Rnd3
D
2.5
2.0
1.5
1.0
0.5
0.0
-1.34
-1.84
-2.34
-2.84
-3.34
mm posterior to bregma

FIGURE S2

A

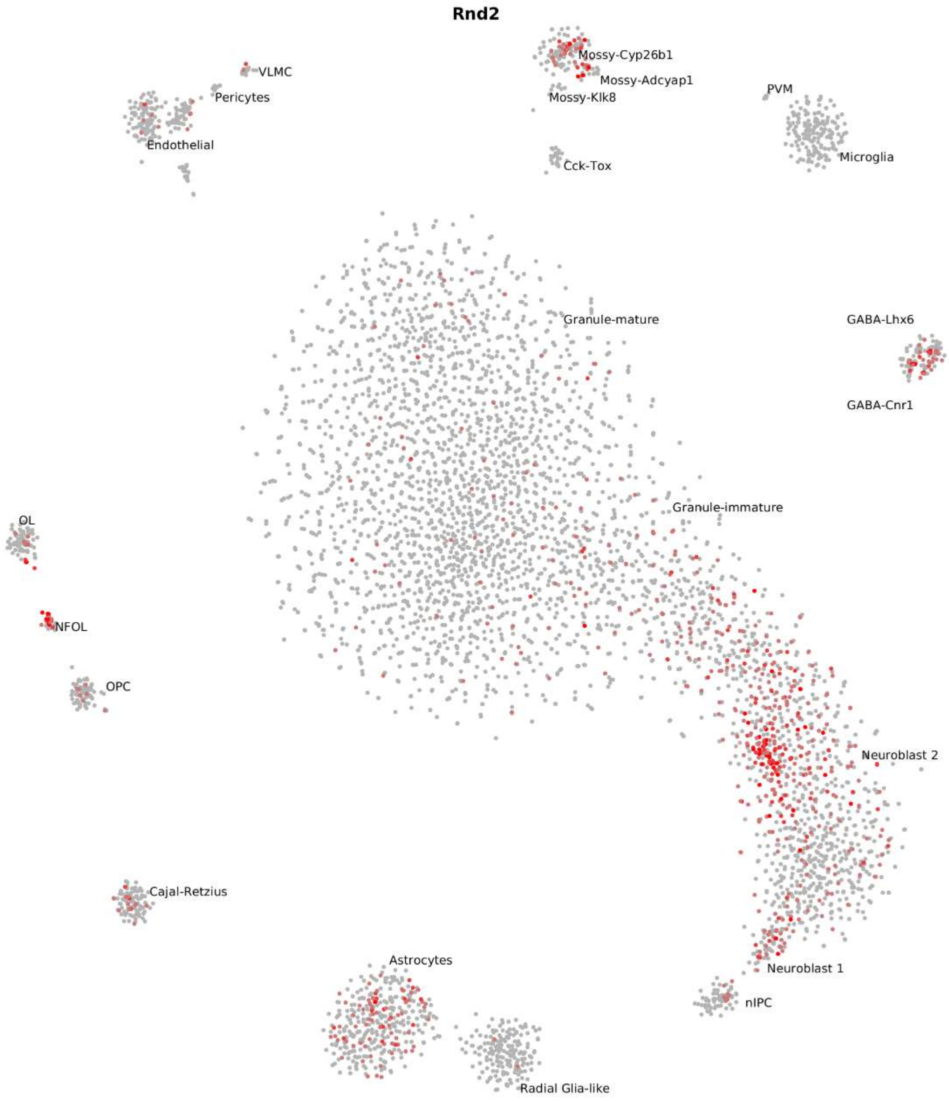
Rnd2
VLMC
Pericytes
Endothelial
Mossy-Cyp26b1
Mossy-Adcyap1
Mossy-Klk8
Cck-Tox
PVM
Microglia
Granule-mature
GABA-Lhx6
GABA-Cnr1
Granule-immature
OL
NFOL
OPC
Neuroblast 2
Cajal-Retzius
Astrocytes
Neuroblast 1
nIPC
Radial Glia-like

FIGURE S3

A

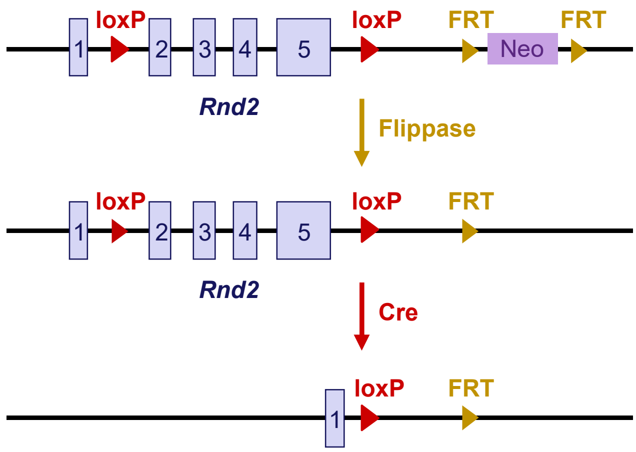

loxP
loxP
FRT
FRT
1
2
3
4
5
Neo
Rnd2
Flippase
Cre

B

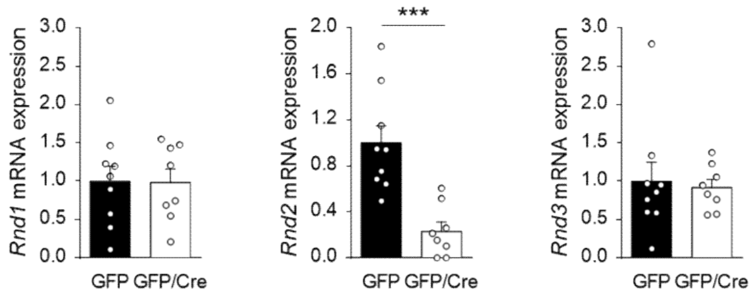

Rnd1 mRNA expression
Rnd2 mRNA expression
Rnd3 mRNA expression
***
GFP GFP/Cre
GFP GFP/Cre
GFP GFP/Cre

FIGURE S4

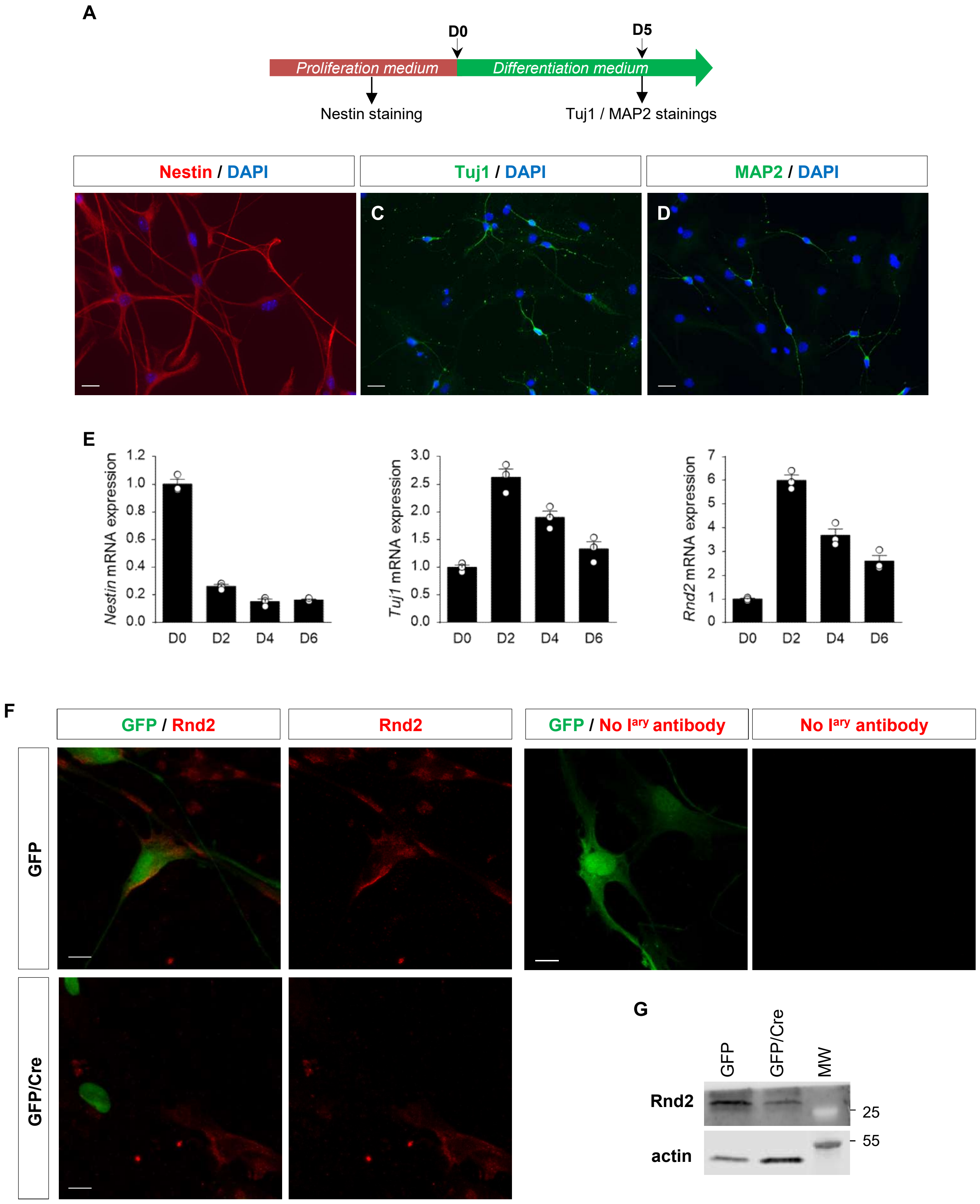

A
D0
D5
Proliferation medium
Differentiation medium
Nestin staining
Tuj1 / MAP2 stainings
Nestin / DAPI
Tuj1 / DAPI
MAP2 / DAPI
C
D
E
Nestin mRNA expression
Tuj1 mRNA expression
Rnd2 mRNA expression
D0
D2
D4
D6
F
GFP / Rnd2
Rnd2
GFP / No Iary antibody
No Iary antibody
GFP
GFP/Cre
G
GFP
GFP/Cre
MW
Rnd2
actin
25
55

FIGURE S5

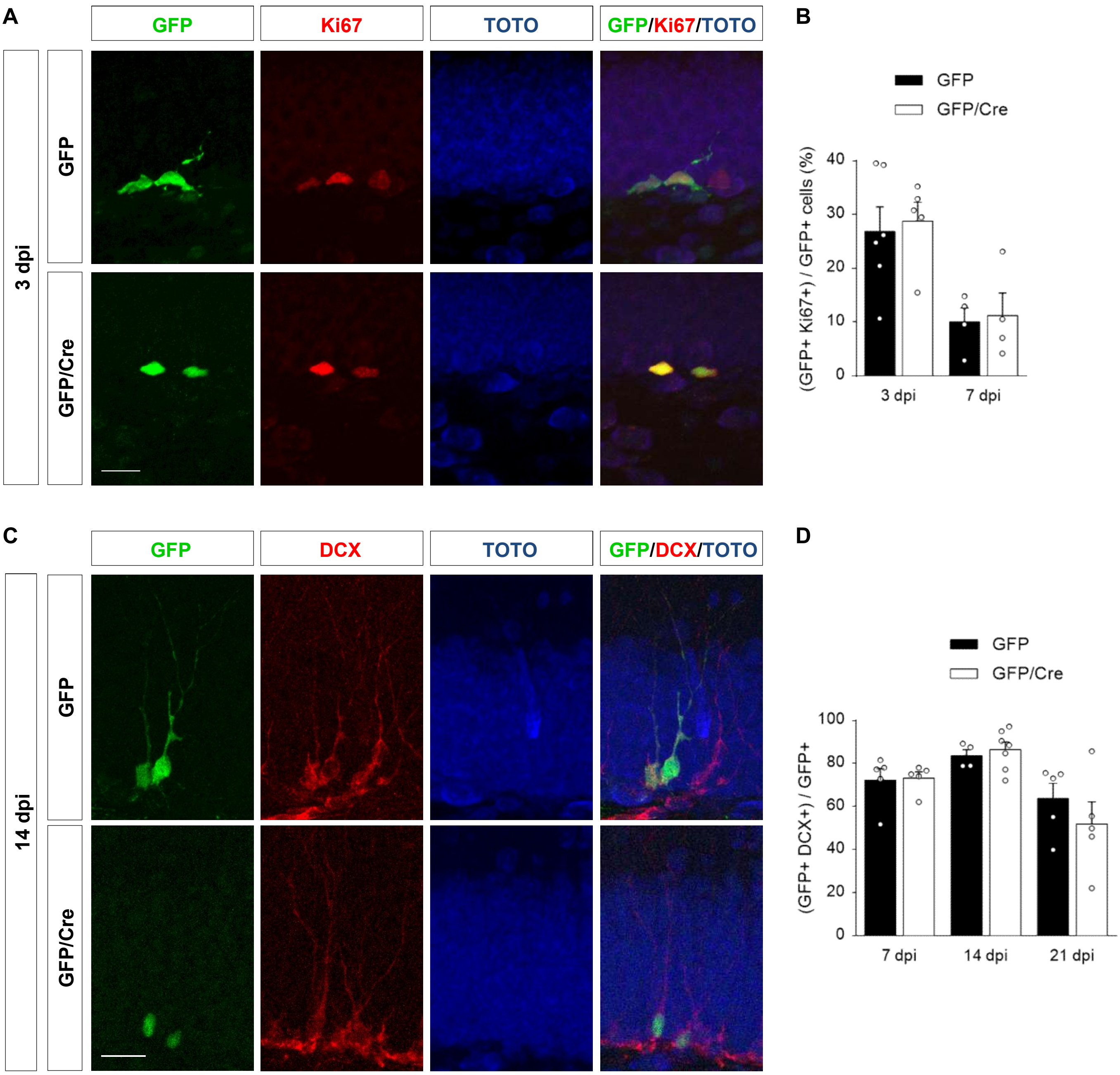
A
GFP
Ki67
TOTO
GFP/Ki67/TOTO
3 dpi
GFP
GFP/Cre
B
GFP
GFP/Cre
(GFP+ Ki67+) / GFP+ cells (%)
40
30
20
10
0
3 dpi
7 dpi
C
GFP
DCX
TOTO
GFP/DCX/TOTO
14 dpi
GFP
GFP/Cre
D
GFP
GFP/Cre
(GFP+ DCX+) / GFP+
100
80
60
40
20
0
7 dpi
14 dpi
21 dpi

FIGURE S6

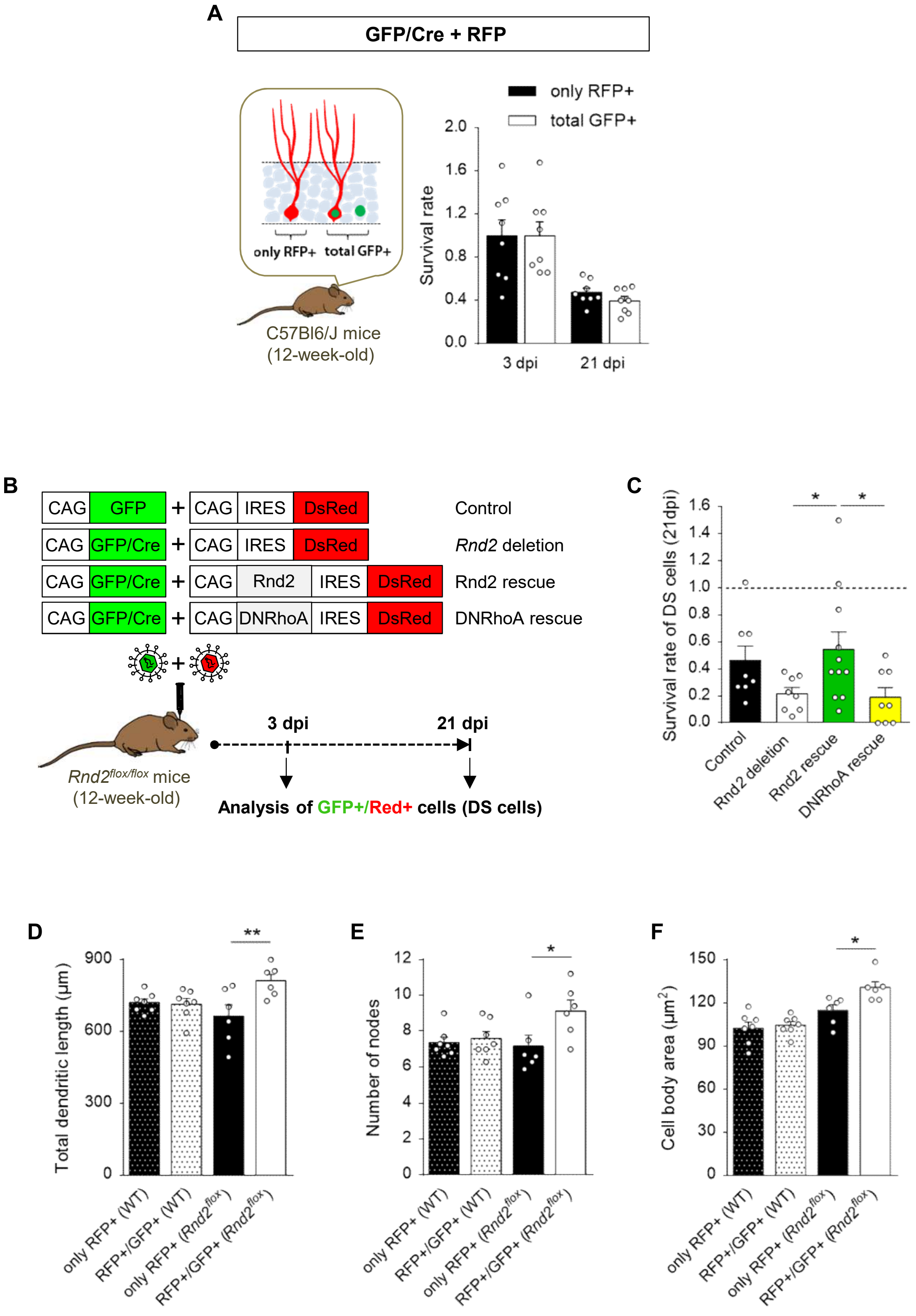
A
GFP/Cre + RFP
only RFP+
total GFP+
C57Bl6/J mice
(12-week-old)
Survival rate
3 dpi
21 dpi
B
CAG GFP + CAG IRES DsRed Control
CAG GFP/Cre + CAG IRES DsRed Rnd2 deletion
CAG GFP/Cre + CAG Rnd2 IRES DsRed Rnd2 rescue
CAG GFP/Cre + CAG DNRhoA IRES DsRed DNRhoA rescue
Rnd2flox/flox mice
(12-week-old)
3 dpi
21 dpi
Analysis of GFP+/Red+ cells (DS cells)
C
Survival rate of DS cells (21dpi)
Control
Rnd2 deletion
Rnd2 rescue
DNRhoA rescue
D
Total dendritic length (µm)
E
Number of nodes
F
Cell body area (µm²)
only RFP+ (WT)
RFP+/GFP+ (WT)
only RFP+ (Rnd2flox)
RFP+/GFP+ (Rnd2flox)

FIGURE S7

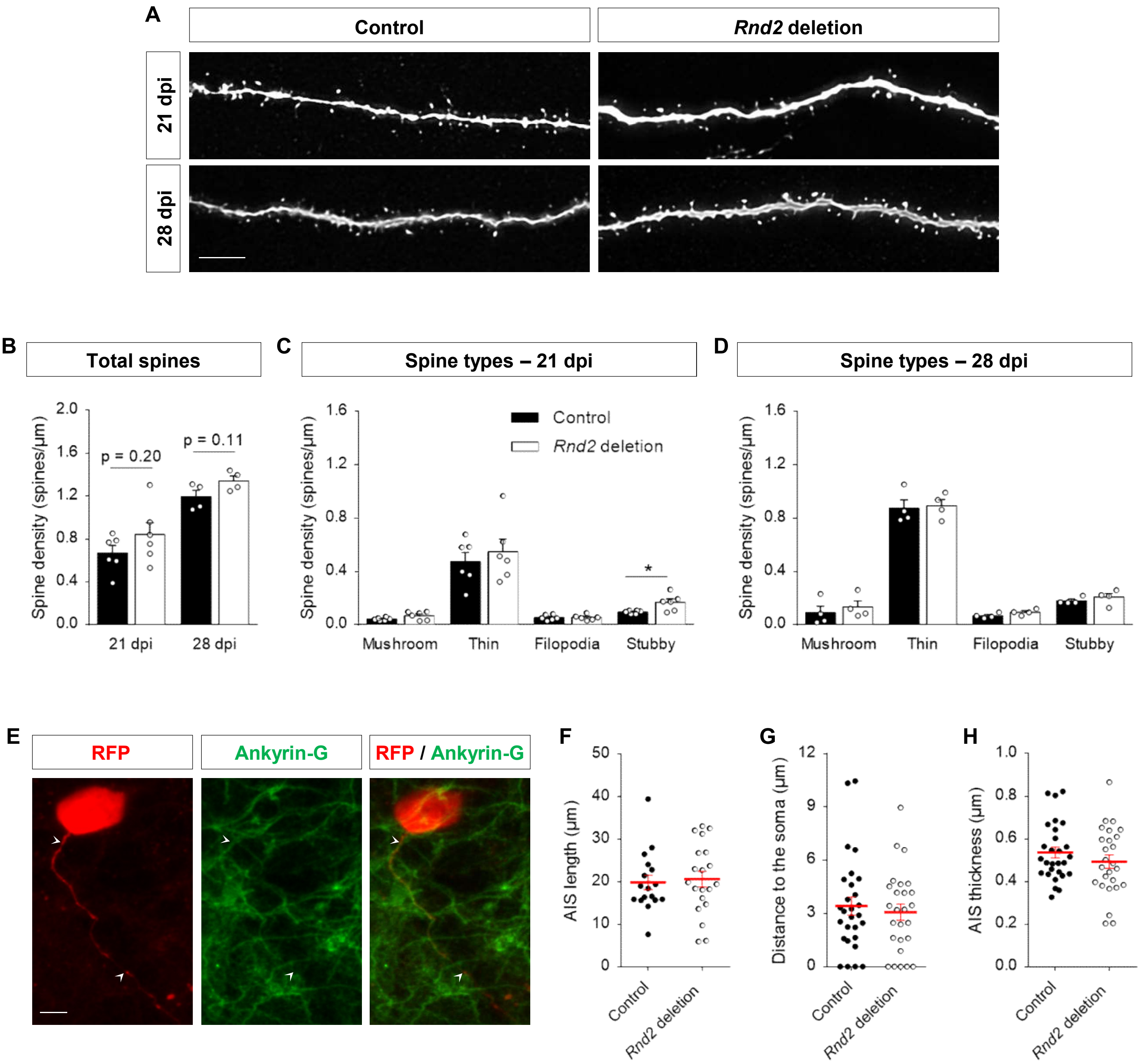

A
Control
Rnd2 deletion
21 dpi
28 dpi
B
Total spines
Spine density (spines/µm)
2.0
1.6
1.2
0.8
0.4
0.0
p = 0.20
p = 0.11
21 dpi
28 dpi
C
Spine types – 21 dpi
Spine density (spines/µm)
1.6
1.2
0.8
0.4
0.0
Control
Rnd2 deletion
*
Mushroom
Thin
Filopodia
Stubby
D
Spine types – 28 dpi
Spine density (spines/µm)
1.6
1.2
0.8
0.4
0.0
Mushroom
Thin
Filopodia
Stubby
E
RFP
Ankyrin-G
RFP / Ankyrin-G
F
AIS length (µm)
50
40
30
20
10
0
Control
Rnd2 deletion
G
Distance to the soma (µm)
12
9
6
3
0
Control
Rnd2 deletion
H
AIS thickness (µm)
1.0
0.8
0.6
0.4
0.2
0.0
Control
Rnd2 deletion

FIGURE S8

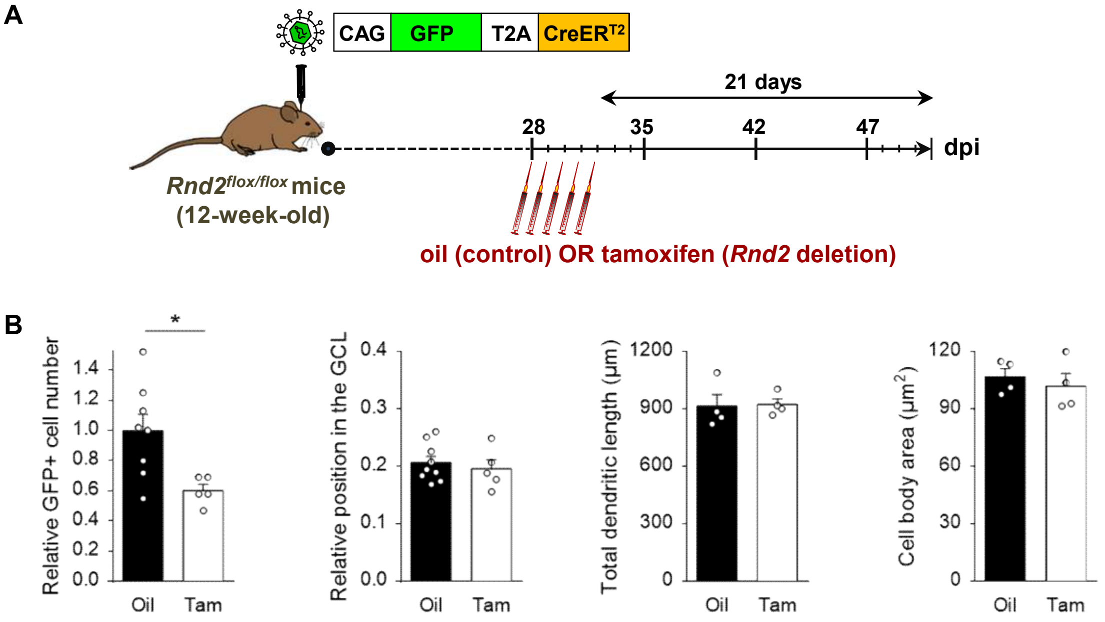
A
CAG
GFP
T2A
CreERT2
21 days
28
35
42
47
dpi
Rnd2flox/flox mice
(12-week-old)
oil (control) OR tamoxifen (Rnd2 deletion)
B
*
Relative GFP+ cell number
Relative position in the GCL
Total dendritic length (μm)
Cell body area (μm2)
Oil
Tam

FIGURE S9

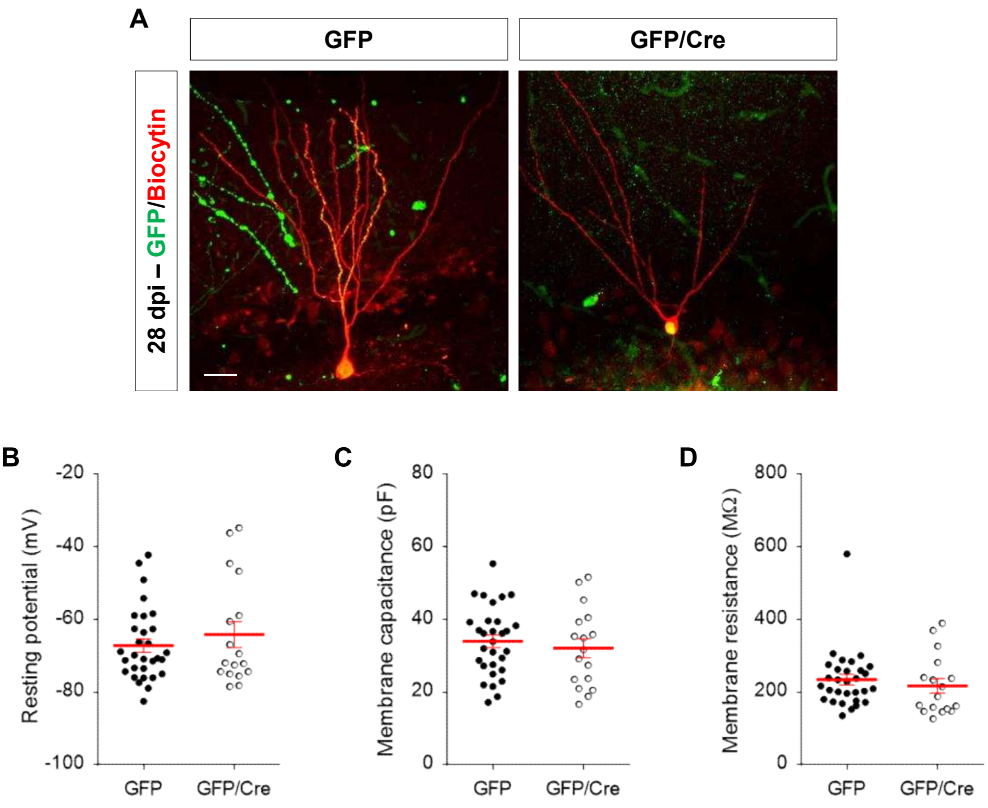
A
GFP
GFP/Cre
28 dpi – GFP/Biocytin
B
Resting potential (mV)
-20
-40
-60
-80
-100
GFP
GFP/Cre
C
Membrane capacitance (pF)
80
60
40
20
0
GFP
GFP/Cre
D
Membrane resistance (MΩ)
800
600
400
200
0
GFP
GFP/Cre

FIGURE S10

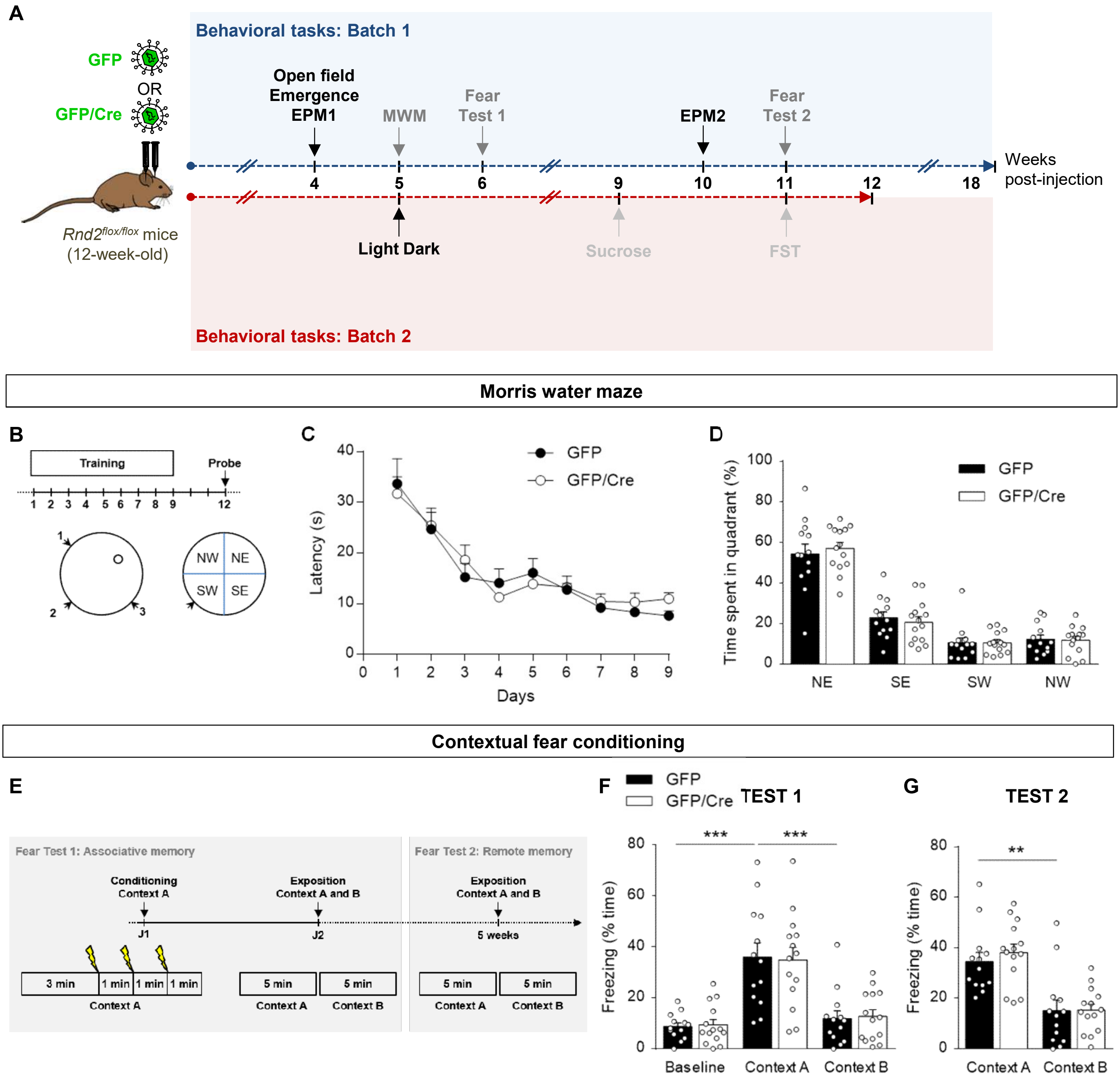
A
Behavioral tasks: Batch 1
GFP
OR
GFP/Cre
Rnd2flox/flox mice
(12-week-old)
Open field
Emergence
EPM1
MWM
Fear
Test 1
EPM2
Fear
Test 2
4
5
6
9
10
11
12
18
Weeks
post-injection
Light Dark
Sucrose
FST
Behavioral tasks: Batch 2
Morris water maze
B
Training
Probe
1 2 3 4 5 6 7 8 9
12
1
2
3
NW
NE
SW
SE
C
GFP
GFP/Cre
Latency (s)
Days
D
Time spent in quadrant (%)
GFP
GFP/Cre
NE
SE
SW
NW
Contextual fear conditioning
E
Fear Test 1: Associative memory
Conditioning
Context A
Exposition
Context A and B
J1
J2
3 min
1 min
1 min
1 min
Context A
5 min
5 min
Context A
Context B
Fear Test 2: Remote memory
Exposition
Context A and B
5 weeks
5 min
5 min
Context A
Context B
F
GFP
GFP/Cre
TEST 1
Freezing (% time)
***
***
Baseline
Context A
Context B
G
TEST 2
Freezing (% time)
**
Context A
Context B

FIGURE S11

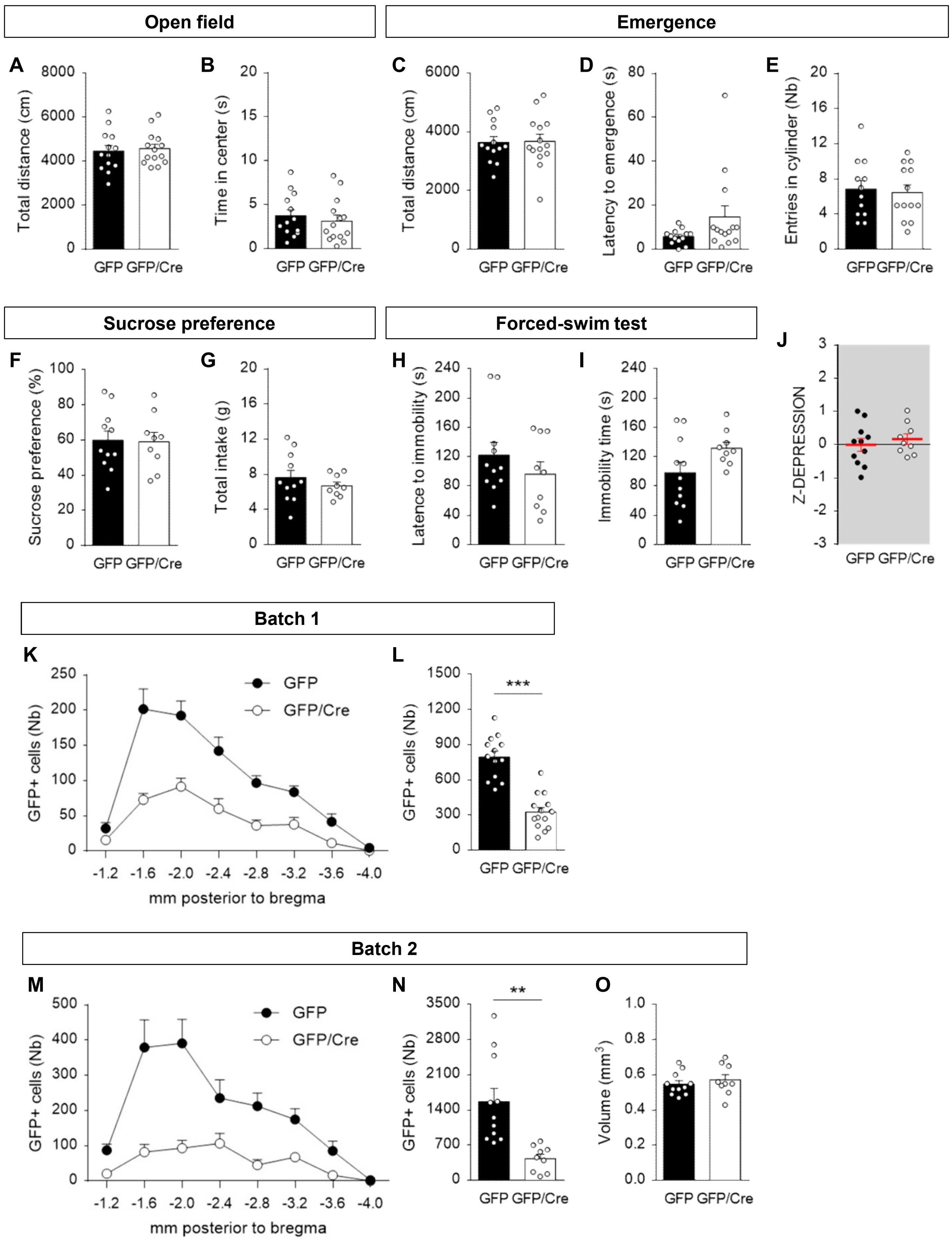

Open field
Emergence
A
Total distance (cm)
B
Time in center (s)
C
Total distance (cm)
D
Latency to emergence (s)
E
Entries in cylinder (Nb)
GFP GFP/Cre
Sucrose preference
Forced-swim test
F
Sucrose preference (%)
G
Total intake (g)
H
Latence to immobility (s)
I
Immobility time (s)
J
Z-DEPRESSION
Batch 1
K
GFP+ cells (Nb)
GFP
GFP/Cre
mm posterior to bregma
L
***
Batch 2
M
N
**
O
Volume (mm3)

FIGURE S12

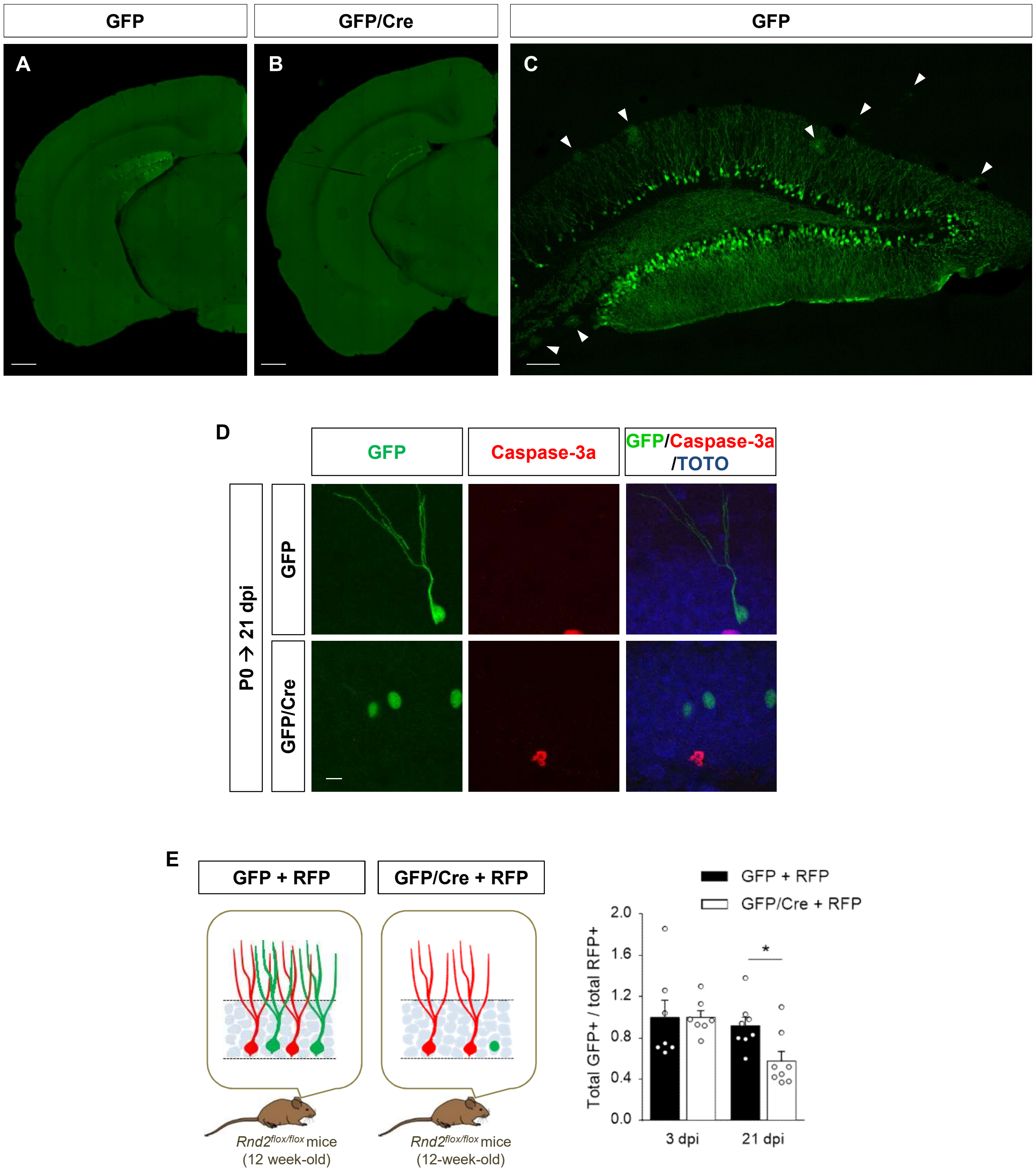

GFP
GFP/Cre
GFP
A
B
C
D
GFP
Caspase-3a
GFP/Caspase-3a
/TOTO
P0 → 21 dpi
GFP
GFP/Cre
E
GFP + RFP
GFP/Cre + RFP
Rnd2flox/flox mice
(12 week-old)
Rnd2flox/flox mice
(12-week-old)
GFP + RFP
GFP/Cre + RFP
Total GFP+ / total RFP+
2.0
1.6
1.2
0.8
0.4
0.0
3 dpi
21 dpi
*

FIGURE S13

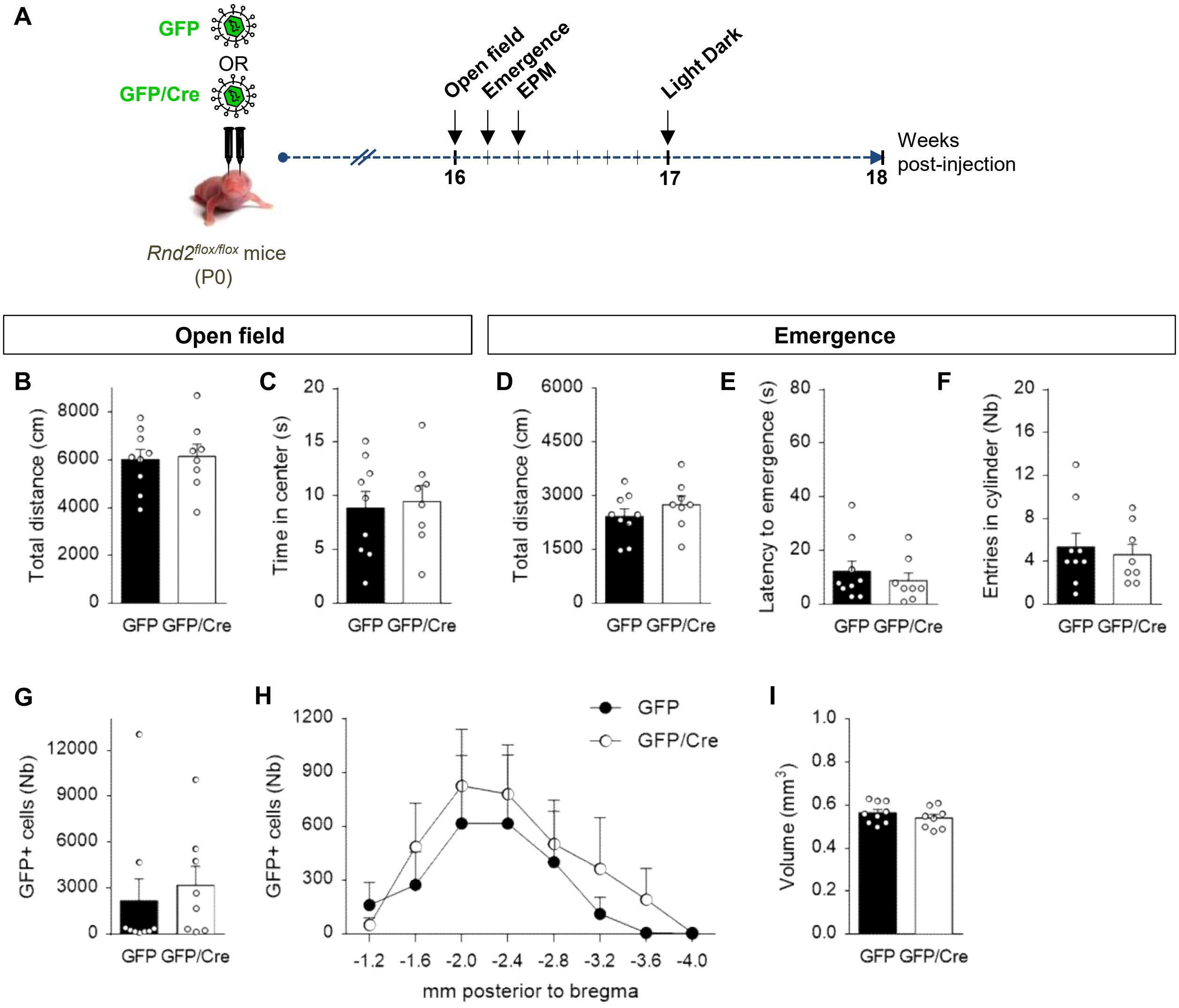
A
GFP
OR
GFP/Cre
Open field
Emergence
EPM
Light Dark
16
17
18
Weeks
post-injection
Rnd2flox/flox mice
(P0)
Open field
Emergence
B
C
D
E
F
Total distance (cm)
Time in center (s)
Total distance (cm)
Latency to emergence (s)
Entries in cylinder (Nb)
GFP GFP/Cre
G
H
I
GFP+ cells (Nb)
GFP+ cells (Nb)
Volume (mm3)
GFP
GFP/Cre
mm posterior to bregma
